# Modulation of the TGF‐β/Smad Signaling Pathway by a Bioactive Oxidized *Polygonati Rhizoma* Polysaccharide Crosslinked Chitosan Hydrogel as Therapeutic Carrier for Parkinson's Disease Treatment

**DOI:** 10.1002/advs.77842

**Published:** 2026-09-30

**Authors:** Peng Dai, Jikuang Zhao, Chang Xue, Kailei Xu, Liang Yong, Zhuangwei Zhang, Yi Huang, Jianwei Shuai, Xianzhen Chen, Sheng Nie, Junpeng Xu

**Affiliations:** ^1^ Central Laboratory of the Medical Research Center The First Affiliated Hospital of Ningbo University Ningbo Zhejiang China; ^2^ UCAS Wenzhou Institute Wenzhou Medical University Wenzhou Zhejiang China; ^3^ National Institute for Data Science in Health and Medicine Xiamen University Xiamen Fujian China; ^4^ Ningbo Key Laboratory of Nervous System and Brain Function, Neurosurgery Department The First Affiliated Hospital of Ningbo University Ningbo Zhejiang China; ^5^ State Key Laboratory of Macromolecular Drugs and Large‐scale Preparation School of Pharmaceutical Science Wenzhou Medical University Wenzhou Zhejiang China; ^6^ Center for Medical and Engineering Innovation Central Laboratory The First Affiliated Hospital of Ningbo University Ningbo Zhejiang China; ^7^ Laboratory of Stem Cell The First Affiliated Hospital of Ningbo University Ningbo Zhejiang China; ^8^ Department of Neurosurgery Shanghai Tenth People's Hospital of Tongji University Shanghai China; ^9^ Cixi People's Hospital Wenzhou Medical University Ningbo Zhejiang China

**Keywords:** bioactive hydrogel, carboxymethyl chitosan, inulin‐type fructan, Parkinson's disease, *Polygonati rhizoma* polysaccharide, TGF‐β signaling pathway

## Abstract

Parkinson's disease (PD) is characterized by progressive dopaminergic neuron loss compounded by oxidative stress and neuroinflammation within a self‐perpetuating pathological brain microenvironment. Here, we report a bioactive injectable self‐healing hydrogel (COPRP) constructed by crosslinking oxidized *Polygonati rhizoma* polysaccharide (OPRP) with carboxymethyl chitosan via dynamic Schiff base linkages. OPRP is structurally identified as an inulin neoseries‐type fructan with relevance to TGF‐β/Smad pathway regulation. Without levodopa loading, COPRP reduced ROS accumulation and M1‐associated inflammatory markers in LPS‐stimulated microglia. It also improved cell viability, preserved mitochondrial membrane potential, and maintained TH expression in 6‐OHDA‐challenged SH‐SY5Y cells, supporting the intrinsic bioactivity of the hydrogel matrix. In a 6‐OHDA rat PD model, COPRP significantly ameliorated motor deficits and preserved TH‐positive neurons. Incorporating levodopa into COPRP creates a dual‐mechanism platform combining active microenvironmental remodeling with passive dopaminergic supplementation. Proteomic analysis, corroborated by western blotting, identified the TGF‐β/Smad signaling axis as the principal mechanistic mediator. Pharmacological intervention with pirfenidone attenuated TGF‐β2/Smad2/3 activation and partially reduced the behavioral and neuroprotective effects associated with COPRP, supporting the functional involvement of this pathway. This work delineates how a defined polysaccharide structure modulates the intracranial pathological microenvironment, offering insights for the rational design of bioactive biomaterials targeting neurodegenerative disease.

## Graphical abstract

An injectable, self‐healing COPRP hydrogel integrates structurally defined oxidized Polygonati rhizoma polysaccharide with carboxymethyl chitosan through dynamic Schiff‐base crosslinking. The bioactive matrix scavenges reactive oxygen species, suppresses neuroinflammation, protects dopaminergic neurons, and supports sustained levodopa release. Its therapeutic effects involve functional activation of TGF‐β2/Smad2/3 signaling within the Parkinsonian brain microenvironment. This design couples intrinsic biomaterial activity with complementary dopaminergic supplementation.

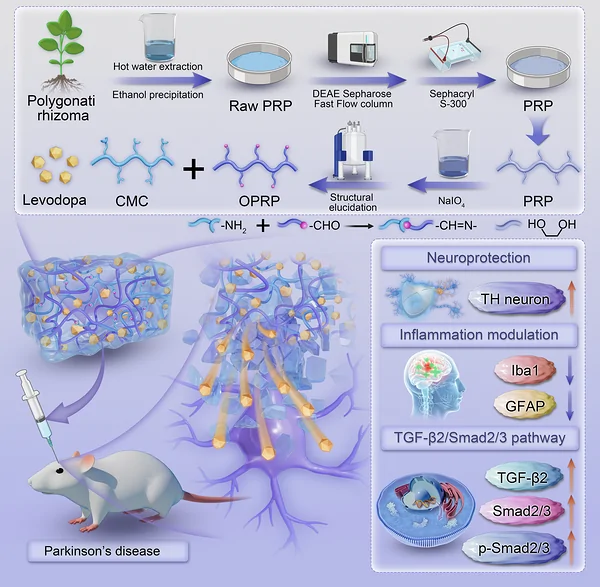

## 1 Introduction

Parkinson's disease (PD) is a common neurodegenerative disorder whose incidence increases significantly with age, severely affecting patients' motor function and quality of life [1]. The primary pathological feature of PD is the progressive loss of dopaminergic neurons in the substantia nigra pars compacta, which leads to a marked decrease in dopamine levels in the brain and consequently gives rise to characteristic symptoms of PD, like bradykinesia and tremor [2, 3]. Furthermore, due to the weakened antioxidant capacity in PD patients, reactive oxygen species (ROS) accumulate in the brain, which in turn triggers oxidative stress that exacerbates mitochondrial dysfunction, lipid peroxidation, and neuronal apoptosis, ultimately leading to profound loss of dopaminergic neurons [4, 5]. Currently available clinical drugs for PD, for example dopamine receptor agonists and monoamine oxidase B inhibitors, although effective in alleviating motor symptoms by modulating dopamine levels and function in the basal ganglia, may cause side effects such as impulse control disorders and hallucinations [6, 7]. Surgical treatments, particularly deep brain stimulation, are limited in application due to high surgical costs [8]. Given the limitations of the aforementioned therapeutic approaches, naturally derived polysaccharides, with excellent biosafety, cost‐effectiveness, and multi‐targeted pharmacological activities, such as antioxidant, anti‐inflammatory, and anti‐apoptotic effects, have emerged as a highly promising class of candidate molecules in the development of PD therapeutics [9, 10].

*Polygonati rhizoma* polysaccharide (PRP), a naturally extracted polysaccharide and the most abundant bioactive component in the rhizome of *Polygonatum sibiricum* (Huangjing), exhibits low toxicity and potent antioxidant/anti‐inflammatory properties, thus holding promising application prospects in the treatment of PD [11, 12, 13, 14]. Huang et al. [15] demonstrated that PRP not only significantly ameliorated motor activity deficits and dopaminergic neuron loss in a 1‐methyl‐4‐phenyl‐1,2,3,6‐tetrahydropyridine‐induced mouse model of PD but also suppressed N‐methyl‐4‐phenylpyridinium‐induced oxidative stress and neuronal apoptosis in vitro. Furthermore, studies have shown that PRP can inhibit neuroinflammation induced by excessive microglial activation and regulate microglial M1/M2 polarization [16]. These findings indicate that PRP possesses neuroprotective potential for the treatment of PD. However, PRP consists predominantly of monosaccharides, e.g., glucose and fructose, and the abundant hydroxyl groups on its molecular chains tend to generate strong intramolecular hydrogen bonds, leading to low reactivity in aqueous solution and difficulty in effectively combining with other drugs or biomaterials, thereby restricting its direct application in drug delivery and tissue engineering.

To overcome the insufficient reactivity and limited applicability of natural polysaccharides, chemical oxidation modification strategies have been widely adopted [17, 18, 19, 20]. Li et al. [21] employed sodium periodate to oxidize hydroxyl groups in dextran into aldehyde/carbonyl groups and utilized the formed aldehydes for further bonding with amino groups to form a hydrogel for bioapplications. Similarly, other oxidized polysaccharides such as oxidized alginate and oxidized hyaluronic acid have been successfully utilized to construct hydrogels for drug or cell delivery [18, 19, 22, 23]. Inspired by this, the controlled oxidized PRP (OPRP) holds promise for yielding a new polysaccharide derivative that possesses both pharmacological and reactive properties for hydrogel preparation. Hydrogels are a class of polymeric materials characterized by a highly hydrophilic three‐dimensional network structure, which can be crosslinked through mechanisms such as intermolecular hydrogen bonding or Schiff base reactions [24, 25, 26]. Among these, self‐healing hydrogels based on the Schiff base reaction (a reversible condensation between an aldehyde group and an amino group) have attracted widespread attention due to the simple and mild reaction conditions, the ability to mimic the microenvironment of tissue repair in vivo, and possession of specific biofunctions [24, 27, 28, 29]. Previous study has proved that a hydrogel prepared by crosslinking oxidized tannic acid‐modified gold nanoparticles with carboxymethyl chitosan (CMC) exhibits favorable injectability, self‐healing properties, and sustained release characteristics [30, 31]. This demonstrates that smart hydrogels based on the Schiff base reaction not only exhibit inherent bioactivities but also serve as delivery vehicles for the localized sustained release of therapeutic agents. Furthermore, utilizing the hydrogels as sustained‐release delivery systems for clinical anti‐PD drugs is a highly promising therapeutic strategy for PD.

Levodopa has been the gold‐standard pharmacotherapy for PD for over 50 years [32, 33]. Levodopa, once converted into dopamine in the brain, is capable of replenishing the reduced dopamine levels caused by the degeneration of dopaminergic neurons in PD patients, thereby alleviating motor symptoms [34]. In the present study, an injectable, self‐healing bioactive hydrogel (COPRP) was designed and fabricated by crosslinking OPRP with CMC via Schiff base linkages (Figure 1). Controlled periodate oxidation introduced reactive aldehyde groups into OPRP. NMR, FTIR, methylation, and monosaccharide analyses identified OPRP as an inulin neoseries‐type fructan. Similar fructan‐rich polysaccharides have been associated with TGF‐β/Smad regulation in other biological contexts. Critically, the independently demonstrated bioactivity of the drug‐free COPRP hydrogel, its capacity to scavenge ROS, suppress microglial neuroinflammation, and protect dopaminergic neuron‐like cells, is attributed to the intrinsic pharmacological properties of the OPRP backbone itself rather than to any exogenous payload. This distinction is central to the conceptual framework of the present work: levodopa loading into COPRPL is not a simple drug carrier relationship, but a deliberate dual‐mechanism design in which OPRP‐mediated active remodeling of the pathological brain microenvironment is complemented by dopaminergic supplementation through levodopa loading. To evaluate this strategy in vivo, COPRP hydrogel was stereotaxically administered into the substantia nigra of 6‐OHDA‐lesioned rats, and therapeutic efficacy was assessed through behavioral, histological, and proteomic analyses. Proteomic profiling followed by western blotting and pharmacological intervention with pirfenidone supported the involvement of the TGF‐β/Smad2/3 signaling pathway through which COPRP orchestrates neuroprotection, resolution of neuroinflammation, and astrocyte phenotype reprogramming in the PD brain. The central scientific contribution of this work therefore lies in elucidating the mechanistic chain linking a defined polysaccharide structural unit, through TGF‐β/Smad pathway activation, to coordinated multi‐target remodeling of the intracranial pathological microenvironment, providing a mechanistically grounded rationale for developing herb‐derived polysaccharide hydrogels as active biological agents in neurodegenerative disease intervention.

**FIGURE 1:**
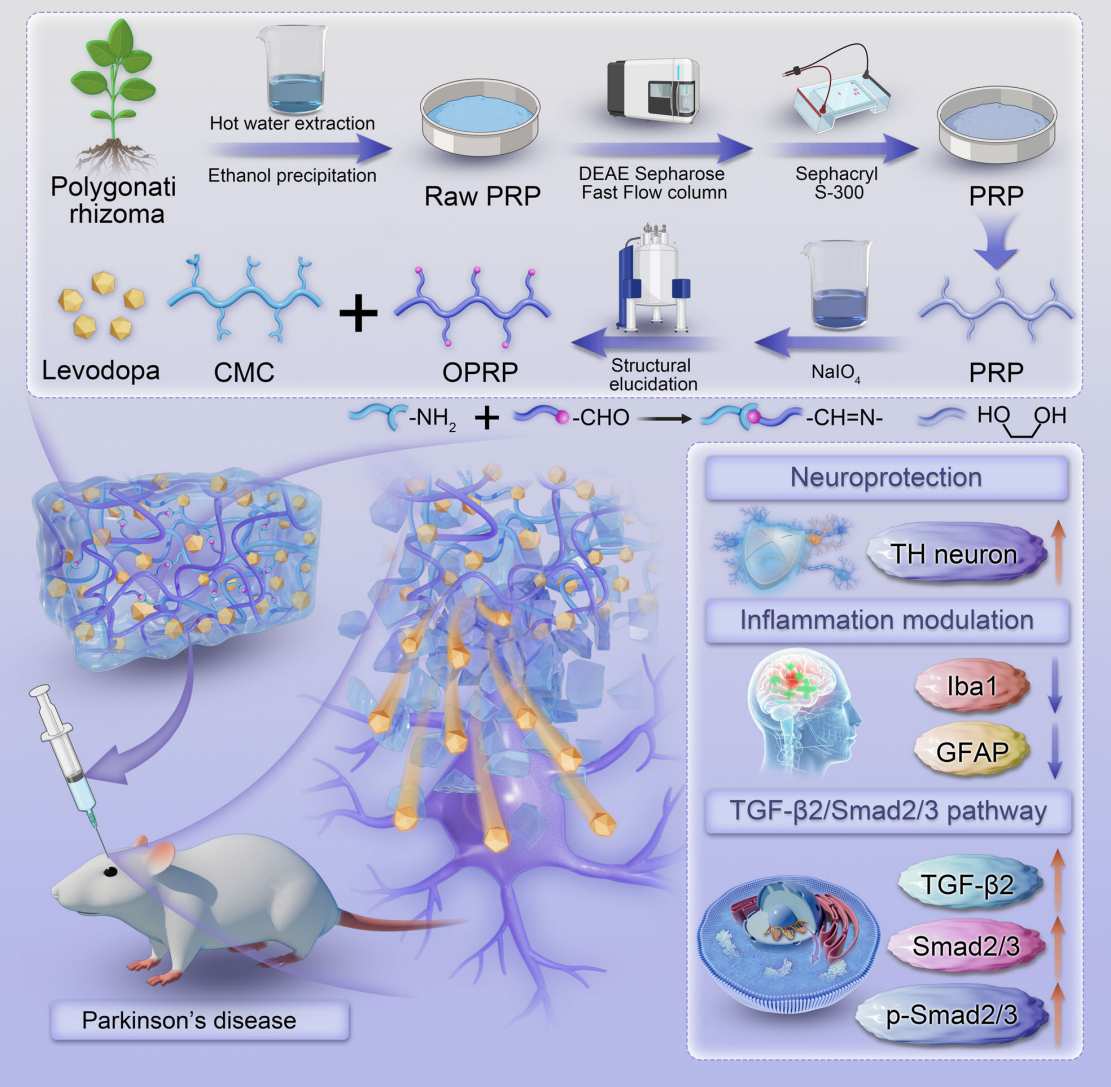
Illustration for the preparation process of oxidized *Polygonati rhizoma* polysaccharide (OPRP) and the gelation process of levodopa‐loaded bioactive self‐healing chitosan hydrogel crosslinking with OPRP and carboxymethyl chitosan (CMC), which possesses sustained drug release, neuroprotective, and anti‐inflammatory functions, for treating Parkinson's disease (PD) by modulating the TGF‐β/Smad signaling pathway, offering a promising strategy for PD therapy.

## 2 Materials and Methods

### 2.1 Synthesis and Structural Elucidation of Oxidized *Polygonati Rhizoma* Polysaccharide (OPRP)

OPRP was synthesized via sodium periodate (NaIO_4;_ 99.5%, Macklin, China) oxidation. PRP was purified from the rhizome of *Polygonatum sibiricum* (Huangjing; Dongbei East Group Co., Ltd., Anhui Province, China), with specific procedures detailed in the preceding work, following slightly modification [11, 13]. Initially, 1 g of PRP was dissolved in 80 mL of deionized water (DI water, H_2_O) and stirred at room temperature until complete homogenization. Subsequently, 656 mg of NaIO_4_ was added to the reaction mixture. After continuous stirring at room temperature for 24 h, the reaction was quenched with 342 µL of ethylene glycol (98%, Macklin, China) for 1 h. The reaction solution was then purified via dialysis using a membrane (MWCO 1000 Da, Yuanye, China) to remove unreacted monomers. Finally, the final product OPRP was obtained by lyophilization of the dialysate. The chemical structure of OPRP was characterized by H Nuclear Magnetic Resonance Spectra (^1^H‐NMR; QUANTUM‐I‐400 MHz, Zhongke Oxford University, China) and Fourier transform infrared spectroscopy (FTIR; Nicolet is50, Thermo Fisher, USA). Structural elucidation was further performed using two‐dimensional NMR (2D‐NMR) and glycosyl residue analysis.

### 2.2 Preparation, Physico‐Chemical Properties, and Rheological Analysis of Carboxymethyl Chitosan (CMC)/OPRP (COPRP) Hydrogel

Based on the Schiff base reactions, the composite COPRP hydrogel was prepared by mixing CMC solution and OPRP solution. The CMC (Mw 10–20w Da, Substitution degree≥80%, Shifeng Biology, China) was dissolved in PBS buffer with 5 wt.%. The OPRP was dissolved in PBS buffer with 1.25, 2.5, and 5% wt.%. Then, at 25°C, the OPRP solution was added into the CMC solution at a volume ratio of 1:1 under vortex to obtain a homogeneous precursor, resulting in the formation of COPRP hydrogel with simultaneous crosslinking. For the CDBP control, 5 wt.% CMC was mixed with 4 wt.% dibenzaldehyde‐terminated polyethylene glycol [35] at a 1:1 volume ratio and allowed to gel at room temperature under the same conditions.

The internal cross‐sectional porous structure of the COPRP hydrogel was observed using field emission scanning electron microscopy (SEM; SU8010, Hitachi, Japan). In vitro degradation of the hydrogel was estimated by monitoring the residual mass over time. At specified time points, the hydrogels soaked in saline at a constant temperature of 37°C were retrieved, rinsed with deionized water, and then freeze‐dried. The percentage of residual weight was calculated using Equation 1:

(1) $$\mathrm{weight}\;\mathrm{remain}\;\left(\%\right)=\frac{W}{W_{0}}\;\times 100\%$$

where *W_0_* is the initial dry weight of the hydrogel, and *W* is the dry weight of the hydrogel after removal at the specified time.

The porosity was evaluated using an ethanol immersion method. The porosity value was achieved by Equation 2:

(2)

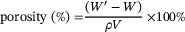

where *W’* is the wet weight of the hydrogel fully swollen in ethanol, *W* is the dried weight of the hydrogel, *ρ* is the density of ethanol, and *V* is the volume of the hydrogel, respectively.

The rheological properties of the COPRP hydrogel were evaluated using a rheometer (DHR‐2, TA Instruments, USA) equipped with a 40 mm‐diameter cone‐plate geometry (2° cone angle) at 25°C. The storage modulus (G′) and loss modulus (G″) were measured as a function of time at a fixed frequency of 1 Hz and a dynamic strain of 1%. Dynamic strain sweep tests were performed at 1 Hz, with the strain amplitude ranging from 1% to 600%. To assess the self‐healing capability of the hydrogel, damage‐healing cycles were conducted by alternately applying high strain (380%) and low strain (1%) at 1 Hz, monitored via rheological measurements. The shear‐thinning behavior of the COPRP hydrogel was characterized by steady shear tests, analyzing the viscosity as a function of shear rate. Finally, the hydrogel was smoothly extruded through a 30G needle with an inner diameter of 0.15 mm and written letters, which demonstrated the macroscopic injectability of the hydrogel.

In vitro drug release experiments of COPRP hydrogels, Fast Green (FCF; 99.95%, New Cell & Molecular Biotech., China) was employed as a hydrophilic model drug for levodopa (Sigma–Aldrich, USA), which has obvious ultraviolet‐visible (UV‐vis) absorption and facilitates concentration tracking in PBS buffer. First, FCF was dissolved in 0.15 mg/mL CMC solution to obtain a final PBS solution of 5 wt.% CMC. Then, COPRP‐FCF hydrogel was prepared as described in Section 2.2 above and placed in a commercial dialysis device (D‐Tube Dialyzer Maxi, MWCO 3.5 kDa, Millipore, USA). Subsequently, the dialysis device was placed in a glass bottle containing 100 mL of PBS buffer at 37°C for dialysis. At predetermined times (0 h, 3 h, 6 h, 12 h, 1 d, 3 d, 5 d, 7 d, 10 d, and 14 d), 1.0 mL of surrounding buffer was removed, and 1.0 mL of fresh buffer was replenished to maintain the total volume of buffer. The release of model drugs was quantified using a UV‐vis spectrometer (Evolution 350, Thermo Fisher, USA). The characteristic peak of FCF was observed at 623 nm.

The free radical scavenging capacity of each experimental group, i.e., CDBP hydrogel, COPRP hydrogel, and levodopa‐loaded COPRP (COPRPL) hydrogel, was assessed using 2,2‐diphenyl‐1‐picrylhydrazyl (DPPH; Aladdin, China). The saline group (Saline) was used as a negative control, and the vitamin C (Vc; Sigma–Aldrich, USA) group was used as a positive control. Absorbance was measured at 517 nm using a UV‐vis spectrometer to quantify the different scavenging activities between the groups.

### 2.3 Scavenging Reactive Oxygen Species (ROS) and Anti‐Inflammatory Experiments

The BV2 mouse microglial cell line was acquired from the Chinese Academy of Sciences Cell Bank (Shanghai, China) and maintained in high‐glucose Dulbecco's Modified Eagle's Medium (DMEM; Gibco, USA) supplemented with 10% fetal bovine serum (FBS; Vivacell, Israel) and 1% penicillin‐streptomycin solution (PS; Beyotime Biotech, China) for routine cell propagation.

ROS scavenging capacity was assessed using a ROS assay kit (Beyotime Biotech, China). After the inflammation of BV2 cells was induced by Lipopolysaccharide (LPS; 10 µg/mL; Sigma–Aldrich, USA) for 24 h, each experimental group was co‐cultured with LPS‐induced BV2 cells for 24 h using a transwell chamber (Costar, USA). Staining was performed at 37°C for 20 min using a ROS‐sensitive fluorescent probe, named DCFH‐DA (Beyotime Biotech, China), and fluorescence images were acquired by inverted fluorescence microscopy (Axio Observer 7, Zeiss, Germany). The fluorescence intensity was quantitatively analyzed using ImageJ software.

The anti‐inflammatory experiments were conducted by culturing BV2 cells in PS‐free DMEM containing 10% FBS to eliminate potential antibiotic interference. BV2 cells were first stimulated with LPS (10 µg/mL) for 12 h to induce M1 polarization. After polarization, each experimental group, including control (unpolarized healthy cells), saline (LPS‐induced cells only), CDBP hydrogel‐treated, COPRP hydrogel‐treated (200 µL), and COPRPL hydrogel‐treated (200 µL) groups, was added to 24‐well plates pre‐inoculated with BV2 cells using a transwell chamber and co‐cultivated for 12 h. The protein expression levels of BV2 cells were further investigated by immunofluorescence (IF) staining and western blotting (WB) analyses.

The IF staining experiments were performed by fixing BV2 cells grown on glass slides with 4 wt.% paraformaldehyde (PFA; Sigma–Aldrich, USA) for 15 min at room temperature after removing the medium and washed three times with PBS for 5 min each, followed by permeabilization with 0.5% Triton X‐100 (Solarbio, China) at room temperature for 15 min. The cells were continued to be washed with PBS three times for 5 min each and then blocked with 5 wt.% bovine serum albumin (BSA; Gibco, USA) solution for 30 min. Next, the BV2 cells were incubated with anti‐IL‐1β antibody (1:200, AF5103; Affinity, UK), anti‐iNOS antibody (1:200, 18985‐1‐AP; Proteintech, USA), anti‐TNF‐α antibody (1:200, AF7014; Affinity, UK) or anti‐CD206 antibody (1:200, DF4149; Affinity, UK) at 4°C overnight. The next day, cell slides were washed with 0.1 wt.% Tween 20 in PBS (PBST) solution and incubated with CoraLite488‐conjugated goat anti‐rabbit IgG(H+L) (1:200, SA00013‐2; Proteintech, USA), or CoraLite594‐conjugated goat anti‐rabbit IgG(H+L) (1:200, SA00013‐4; Proteintech, USA) for 1 h at 25°C. After washing with PBST, the cell nuclei were counterstained with the DAPI staining solution (Beyotime Biotech., China). The photomicrographs were captured with an inverted fluorescence microscope. The semi‐quantitative data for average fluorescent intensity of IL‐1β/iNOS/TNF‐α/CD206‐positive BV2 cells were calculated and acquired using ImageJ software.

The WB experiments were performed by extracting proteins from BV2 cell homogenates and using the bicinchoninic acid (BCA) protein assay kit (WB6501; New Cell & Molecular Biotech., China) to determine protein concentrations according to the manufacturer's protocol. Briefly, proteins were transferred by electrophoresis onto a 0.45 µm polyvinylidene fluoride membrane (PVDF Immobilon‐P, IPVH00010; Millipore, USA). Then, the membrane was blocked with 5% (w/v) skimmed milk (BioFroxx, Germany) in 1 × TBST solution for 2 h. Primary antibodies included anti‐IL‐1β antibody (1:1000, BF8021; Affinity, UK), anti‐CD206 antibody (1:1000, DF4149; Affinity, UK), anti‐TNF‐α antibody (1:1000, AF7014; Affinity, UK), anti‐iNOS antibody (1:1000, AF0199; Affinity, UK) or anti‐β‐tubulin antibody (1:10000, T0023; Affinity, UK). Membranes were incubated overnight at 4°C, washed three times with TBST, and probed with HRP‐conjugated goat anti‐mouse IgG (H+L) secondary antibody (1:1000, A0216; Beyotime Biotech., China) or HRP‐conjugated goat anti‐rabbit IgG (H+L) secondary antibody (1:1000, A0208; Beyotime Biotech., China) for 2 h at room temperature. Protein bands were imaged with a ChemiDoc XRS+Imaging System (Bio‐Rad, USA). Band intensities were quantified using ImageJ software.

### 2.4 In Vitro Cellular Parkinson's Disease (PD) Model Experiments

The SH‐SY5Y (a human neuroblastoma cell line) was purchased from Shanghai Fuheng Biotechnology Co., Ltd. and maintained in DMEM supplemented with 10% FBS and 1% PS for routine cell propagation. To establish an in vitro cellular PD model, SH‐SY5Y cells were first induced with 6‐hydroxydopamine [6‐OHDA; 100 µm in 0.02 wt.% ascorbic acid saline solution (Sigma–Aldrich, USA)] for 24 h. Following induction, experimental groups, including control, saline, CDBP hydrogel, COPRP hydrogel, and COPRPL hydrogel groups (200 µL/well) were added to a 24‐well plate pre‐seeded with SH‐SY5Y cells and co‐cultured for a specified period of time. The therapeutic efficacy of each group on SH‐SY5Y cells was further evaluated through cell viability test, apoptosis detection, mitochondrial membrane potential (MMP) measurements, cellular IF staining, and WB experiments.

Cell viability was assessed using the cell counting kit‐8 (CCK‐8; New Cell and Molecular Biotech., China) after co‐culturing 6‐OHDA‐induced SH‐SY5Y cells with the experimental groups for 3 days. Absorbance measurements were performed at a wavelength of 450 nm using a microplate reader (Multiskan SkyHigh 1550; Thermo Fisher Scientific, USA). The cell survival rates of all hydrogel groups were standardized relative to the control group, defined as cells cultured in medium alone.

The apoptosis of 6‐OHDA‐induced SH‐SY5Y cells co‐cultured with the experimental groups for 24 h was detected using the Annexin V‐FITC/PI Apoptosis Detection Kit (Servicebio, China). First, the cell culture supernatant was collected. The cells were then digested with EDTA‐free trypsin (Beyotime Biotech, China) and combined with the collected supernatant. The mixture was centrifuged at 500 × g for 5 min at 4°C to pellet the cells. Subsequent procedures were performed strictly following the manufacturer's protocol. Finally, fluorescence images were acquired via an inverted fluorescence microscope, and the semi‐quantitative data for average fluorescent intensity of Annexin V‐FITC/PI‐positive SH‐SY5Y cells were calculated and acquired using ImageJ software.

The MMP for 6‐OHDA‐induced SH‐SY5Y cells treated with the experimental groups for 24 h was detected using the MMP detection kit (JC‐1; Beyotime Biotech., China). Operate strictly according to the manufacturer's instructions. First, remove the cell culture medium from the well plate. Add 1 mL of incubation solution prepared by equilibrating JC‐1 staining solution with cell culture medium at a 1:1 volumetric ratio, and mix thoroughly. Incubate at 37°C in a cell culture incubator for 20 min. Subsequent procedures were performed strictly following the manufacturer's protocol. Finally, fluorescence images were acquired via an inverted fluorescence microscope, and the semi‐quantitative data for average fluorescent intensity of Aggregates/Monomers‐positive SH‐SY5Y cells were calculated and acquired using ImageJ software. For the apoptosis and mitochondrial membrane‐potential assays, fluorescence intensity was quantified from three randomly selected, non‐overlapping fields per sample across at least three independent experiments. Images within each assay were acquired using identical magnification, exposure time, and acquisition settings. JC‐1 fluorescence images were acquired using a 40× objective.

The IF of tyrosine hydroxylase (TH)‐positive cells after co‐culturing 6‐OHDA‐induced SH‐SY5Y with the experimental groups for 24 h was observed. For specific experimental procedures, refer to the IF staining experimental steps in Section 2.3 above. The primary antibody used is anti‐TH rabbit monoclonal antibody (1:200, AB137869; Abcam, UK). Secondary antibody used is CoraLite594‐conjugated goat anti‐rabbit IgG(H+L) (1:200, SA00013‐4; Proteintech, USA). The cells nuclei were counterstained with the DAPI staining solution. The photomicrographs were captured with an inverted fluorescence microscope. The semi‐quantitative data for average fluorescent intensity of TH‐positive SH‐SY5Y cells were calculated and acquired using ImageJ software. The TH protein expression was observed via WB experiment after co‐culturing 6‐OHDA‐induced SH‐SY5Y cells with the experimental group for 24 h. For specific experimental procedures, refer to the WB experimental methods described in Section 2.3 above. The primary antibodies included anti‐TH rabbit monoclonal antibody (1:5000, AB137869; Abcam, UK) and anti‐GAPDH rabbit polyclonal antibody (1:10000, AF7021; Affinity, UK). Secondary antibody used is HRP‐conjugated goat anti‐rabbit IgG (H+L) (1:1000, A0208; Beyotime Biotech., China). Protein bands were imaged with a ChemiDoc XRS+Imaging System. Band intensities were quantified using ImageJ software.

### 2.5 In Vivo Rat Model of PD and In Situ Stereotaxic Injection

The animal experiments were performed using adult male Sprague Dawley (SD) rats weighing 200–250 g purchased from Beijing Vitality River Laboratory Animal Technology Co., Ltd. All animal experimental procedures in this study were approved by the Institutional Animal Care and Use Committee (IACUC) of the Oujiang Laboratory (Approval No. OJLAB24122503) and were in accordance with the Animal Care and Use Guidelines Laboratory Animals. Rats were housed in a constant temperature and humidity environment with a 12 h light/dark cycle, with free access to food and water. It was previously reported in the literature that the protocol of inducing PD in rats by microinjection of 6‐OHDA into the unilateral medial forebrain bundle (mfb) [30, 36, 37].

After being anesthetized with veterinary anesthetics (Zoletil 50, dose 40 mg/kg, Virbac, France), the rats were then immobilized in a stereotaxic apparatus (RWD, China). Using a 26‐gauge Hamilton microsyringe (702RN‐25 µL; Hamilton Company, USA), 6‐OHDA (4 µL each, 2 µg/µL in 0.02 wt.% ascorbic acid saline solution) was injected into the mfb. The injection site was selected as anterior‐posterior (AP) ‐2.8 mm × lateral (LAT) ‐2.0 mm × depth (DEP) ‐8.0 mm, and the flow rate was set at 0.5 µL/min, according to Paxinos and Watson's Rat Brain Mapping Coordination [38]. Withdrawing the injection cannula was preceded by an additional 5‐min wait to minimize residual fluid loss along the injection pathway. To assess the lesion effect of the model, the rotational behavior of all rats was examined by additional injections of apomorphine (APO, *R*‐(−)‐Apomorphine hydrochloride hemihydrate, PHR2621, Sigma‐Aldrich, USA; 0.05 mg/kg s.c., 5 mg/mL dissolved in 0.2 wt.% ascorbic acid saline solution). The metric for successful induction of PD was that after injection of APO, the PD rats rotated more than 25 times per 5 min and always turned to the contralateral side of the injury site. The regulatory role of COPRP hydrogels on TGF‐β‐related pathways was investigated using Pirfenidone (Pri; Selleck Chemicals, USA), a pharmacological agent reported to attenuate TGF‐β‐associated signaling, following previous reference [39]. Rats treated with the inhibitor received an intraperitoneal injection of Pri at a dose of 250 mg/kg prior to PD modeling.

Rats with PD successfully induced by behavioral tests were selected for unilateral injection of saline and hydrogel. After anesthetized with Zoletil 50, the heads of rats were fixed to the stereotaxic apparatus (RWD, China). The injection position was consistent with the lesion site. Using a Hamilton microsyringe with a 26‐gauge needle, 4 µL of saline, CDBP hydrogel, COPRP hydrogel, or COPRPL hydrogel was injected at a rate of 0.5 µL/min, respectively. After waiting 5 min for the injection to finish, the needle was withdrawn slowly to prevent reflux. Each experimental group was studied using 8 rats, including behavioral tests, histological analysis, and protein expression assessment. A total of 85 rats were used in this study, including 5 non‐successful model rats and 8 PD rats in each experimental group, for correlation analysis and validation of the TGF‐β/Smad signaling pathway. The success rate of PD modeling in this experiment was approximately 94% (80/85), and there were no animal deaths in PD modeling stage. For better comparison, we employed a sham‐operated (Sham) group, i.e., healthy rats injected with saline instead of 6‐OHDA during the first stage of surgery.

### 2.6 Behavioral Tests of PD Rats

By subcutaneous injection of APO, rotational behavior was assessed at 7 and 14 days after post‐treatment. Rats were placed in a circular open field of 60 cm in diameter, and rotations of 360° toward the contralateral lesion side were recorded over a 5‐min period. Calculation of rotational velocity (s/round) was performed to quantify the attenuation of PD‐related motor asymmetry. Using the innate exploratory behavior of rodents in new environments, forelimb lateralization was assessed using the cylinder test. Rats were individually placed in a 22 cm diameter × 26 cm high clear glass cylinder under dim lighting, and the duration of forelimb contact with the cylinder wall was recorded for 5 min. The number of ipsilateral (impaired) and contralateral (unimpaired) forelimb contacts were calculated as a percentage of the total number of contacts and compared with age‐matched healthy controls. The open field test was conducted to evaluate the spontaneous locomotor and exploratory behaviors of PD rats in an unfamiliar environment, following previously established protocols with minor adaptations [40]. Each rat was placed individually into one corner of a black wooden arena measuring 100 cm in length, 100 cm in width, and 50 cm in height. Locomotor activity was recorded over a 5‑min period using a video camera, and the recordings were subsequently analyzed with an open field behavioral analysis system (Smart 3.0, Panlab SMART video tracking system, Barcelona, Spain). The central zone of the arena was defined as a 50 × 50 cm square located 25 cm from each edge. Between consecutive trials, the floor surface was thoroughly cleaned using a 10% ethanol solution.

### 2.7 Histological Staining, Western Blotting (WB), and Superoxide Dismutase Activity (SOD) of Brain Tissue

In vivo biocompatibility and histological markers of PD were assessed by IF staining. Briefly at 4°C, tissue sections were incubated overnight with primary antibodies: anti‐TH rabbit polyclonal antibody (1:300, GB11181; Servicebio, China) employed to assess dopaminergic neuron integrity, anti‐Iba1 mouse monoclonal antibody (1:500, GB12105; Servicebio, China) utilized to quantify microglial activation, and anti‐glial fibrillary acidic protein (GFAP) rabbit polyclonal antibody (1:1000, GB111096; Servicebio, China) used to evaluate astrocyte reactivity. After rinsing, sections were incubated for 1 h at 25°C with species‐specific secondary antibodies: Cy3‐conjugated goat anti‐rabbit IgG (1:300, GB21303; Servicebio, China), Cy3‐conjugated goat anti‐mouse IgG (1:300, GB21301; Servicebio, China), and Alexa Fluor 488‐conjugated goat anti‐rabbit IgG (1:400, GB25303; Servicebio, China). Fluorescence micrographs were acquired via a digital slide scanner (Pannoramic MIDI, 3DHISTECH Ltd., Hungary), and mean fluorescence intensity was quantified across anatomically defined regions of interest using ImageJ software. As for hematoxylin and eosin (H&E) staining (standard type; Jiangsu KeyGen Biotech Co., Ltd., China), the staining was operated according to the manufacturer's instructions. Brain tissue sections from the sham operation group, saline group, and COPRP group were tested. At the same time, the hearts, livers, spleens, lungs, and kidneys of rats in each group were harvested and stained at the end of the experiment. The H&E‐stained sections were visualized under an optical microscopy (Olympus, Japan).

In the tissue WB experiment, the extracted brain tissues were homogenized, and the homogenate was centrifuged at 12 000 × g for 15 min at 4°C, and the supernatant was collected to quantify total proteins by BCA assay. The remaining steps followed the protocol outlined in Section 2.3. Membranes were probed with the following primary antibodies: anti‐β‐Actin rabbit polyclonal antibody (∼42 kDa, 1:5000, AF7018; Affinity, UK), anti‐GFAP mouse monoclonal antibody (∼50 kDa, 1:1000, BF8023; Affinity, UK), anti‐TGF‐β1 mouse monoclonal antibody (∼45 kDa, 1:1000, AF1027; Affinity, UK), anti‐GAPDH rabbit polyclonal antibody (∼36 kDa,1:10000, AF7021; Affinity, UK), anti‐TGF‐β2 mouse monoclonal antibody (∼47 kDa, 1:1000, AF0260; Affinity, UK), anti‐Smad2/3 rabbit polyclonal antibody (∼60 kDa, 1:1000, AF6367; Affinity, UK), anti‐p‐Smad2/3 rabbit polyclonal antibody (∼60 kDa, 1:500, AF3367; Affinity, UK). HRP‐conjugated secondary antibodies included goat anti‐rabbit IgG (H+L) (1:5000, A0208; Beyotime Biotech., China) and goat anti‐mouse IgG (H+L) (1:5000, A0216; Beyotime Biotech., China). Protein bands were visualized using the ChemiDoc XRS+Imaging System and quantified via ImageJ software. Detection of superoxide dismutase (SOD) activity was performed using the Total Superoxide Dismutase Assay Kit with WST‐8 (Beyotime Biotech., China), adhering strictly to the manufacturer's protocol. Absorption values were acquired using a microplate reader at a wavelength of 450 nm. All data were standardized based on tissue weight (mg) to correct for regional variations in protein content.

### 2.8 Proteomics Analysis

#### 2.8.1 Sample Preparation

Proteomic analysis was performed on 6 rat brain tissue samples (including 3 samples from the CDBP group and 3 samples from the COPRP hydrogel group). Sample preparation involved the following steps: protein extraction, denaturation, reduction, alkylation, tryptic digestion, and peptide cleanup. This procedure was performed using the commercially available iST sample preparation kit (PreOmics, Germany) in strict accordance with the manufacturer's instructions. In brief, 50 µL of lysis buffer was added to each sample, followed by heating at 95°C for 10 min under continuous agitation at 1000 rpm. After allowing the samples to cool to room temperature, trypsin digestion buffer was added, and the mixture was incubated at 37°C for 2 h with shaking at 500 rpm. The enzymatic digestion was terminated by the addition of stop buffer. Subsequently, the resulting peptides were purified and desalted using the iST cartridge with the recommended wash buffers. Finally, the purified peptides were eluted twice with 100 µL of elution buffer each time and then concentrated to dryness using a SpeedVac centrifuge.

#### 2.8.2 DIA Data Acquisition

The desalted and lyophilized peptide samples were reconstituted in mobile phase A (0.1% formic acid in water) and subsequently analyzed by LC‐MS/MS [41, 42]. Liquid chromatography‐mass spectrometry (LC‐MS) analysis was carried out using an UltiMate 3000 LC system (Thermo Fisher Scientific, MA, USA) coupled to a timsTOF HT mass spectrometer (Bruker Daltonik, Bremen, Germany), which is an ion mobility spectrometry quadrupole time‐of‐flight instrument. The peptide samples were redissolved in 0.1% formic acid (FA), and an aliquot of 200 ng of peptides was loaded onto an AUR3‐15075C18 column (15 cm length, 75 µm internal diameter, 1.7 µm particle size, 120 Å pore size; IonOpticks). Chromatographic separation was achieved using a 30‐min gradient elution program starting with 4% mobile phase B (80% acetonitrile containing 0.1% FA), which was then increased stepwise to 28% over 25 min, further raised to 90% within 1.5 min, and finally held at 80% for 3.5 min. The flow rate was maintained at 300 nL/min, and the column temperature was kept at 50°C. Data‐independent acquisition (DIA) was performed in the diaPASEF mode. A total of 24 consecutive precursor isolation windows, each 25 Th wide, were defined across the m/z range of 400–1000. To optimize the MS1 cycle time, three repetitions per step were set within an 8‐scan diaPASEF scheme. During PASEF MS/MS scanning, the collision energy was linearly ramped as a function of ion mobility, ranging from 59 eV at 1/K_0_ = 1.6 Vs/cm^2^ to 20 eV at 1/K_0_ = 0.6 Vs/cm^2^.

#### 2.8.3 Database Search

Raw DIA data were processed and analyzed using Spectronaut 19 (Biognosys AG, Switzerland) under default parameter settings. A protein sequence database for Rattus norvegicus (Version 2025, containing 22,369 entries) was downloaded from UniProt and used for the search. Trypsin was specified as the digestive enzyme, with a strict digestion mode. Carbamidomethylation of cysteine residues was set as a fixed modification, while oxidation of methionine and N‐terminal acetylation of proteins were included as variable modifications. Retention time prediction was performed using the dynamic iRT approach. Spectronaut conducted data extraction based on comprehensive mass calibration and automatically determined the optimal extraction window according to iRT calibration and gradient stability. False discovery rates (FDRs) for precursor, peptide, and protein identification were all controlled at 1%, as determined by Q‐value cutoffs. The decoy database was generated using the mutated strategy, which resembles the scrambled method but involves random swapping of a variable number of amino acid positions (minimum of 2 swaps and maximum of half the peptide length). For normalization, the local normalization strategy was applied. Peptides meeting the 1% Q‐value threshold were used to quantify protein groups via the MaxLFQ algorithm.

### 2.9 Statistical Analysis

Quantitative data were acquired from independently repeated experiments, and all experiments had at least 3 biological replicates. Results are expressed as mean ± standard deviation (STD). Statistical significance was assessed by one‐way analysis of variance (ANOVA) and Tukey's post‐hoc test (α = 0.05) for comparisons between multiple cohorts. Behavioral data collected at multiple time points within the same cohort were analyzed by two‐way repeated‐measures ANOVA to account for time‐ and treatment‐dependent effects, and pairwise comparisons were performed using Tukey's post‐hoc test. The statistical analyses were performed using GraphPad Prism 9.5. A *p*‐value less than 0.05 was indicated by an asterisk in the figures and was considered statistically significant.

## 3 Results and Discussion

### 3.1 Synthesis and Structural Elucidation of OPRP

The synthesis of OPRP is based on modifications and integration of previous work and follows a two‐step procedure [17, 18, 19, 43]. PRP has been preliminarily confirmed to consist of fructose and glucose, featuring a molecular chain rich in diols [11, 13, 44]. Under neutral aqueous conditions, NaIO_4_ can oxidatively cleave these vicinal diol structures in PRP to yield corresponding aldehyde groups [21], as shown in the synthetic route in Figure 1. The ^1^H‐NMR spectra of PRP and OPRP are shown in Figure S1 and Figure 2A. Aldehyde proton was expected between 9.0 and 9.5 ppm in the NMR spectrograph of OPRP, which is not observed however [45, 46]. The omission of aldehyde proton signals suggests that aldehyde groups reacted with adjacent hydroxyl groups to form hemiacetals or hemiacetal‐like structures. The multiple peaks observed between 4.0 and 5.5 ppm provide further evidence that hemiacetal groups were indeed formed during the reaction [46]. The FTIR spectrum of PRP and OPRP (Figure 2B) indicated typical characteristic peaks of polysaccharide around ∼3270 (O‐H stretching vibration), ∼2932 (C‐H stretching vibration), ∼1417 (C‐H bending vibration), and ∼1361 cm^−1^ (C‐O bending vibration). The absorption peaks between 1000 and 1250 cm^−1^ correspond to the stretching vibrations of the C‐O‐C group, while the pyranose exhibits three strong absorption peaks in this range, whereas the furanose shows only two [47]. Two absorption peaks within the 1000–1250 cm^−1^ range at 1016 and 1115 cm^−1^, indicating the presence of a furanose structure. Further absorption peaks at 939 and 816 cm^−1^ confirmed the presence of β‐D‐furanose fructose [48, 49, 50]. Meanwhile, the FTIR spectrum reveals the characteristic absorption peak of the C = O bond (1730 cm^−1^) in the OPRP (Figure 2B). These results indicate the successful synthesis of OPRP. The degree of oxidation, determined via hydroxylamine hydrochloride titration, was calculated to be approximately 16.33%.

**FIGURE 2:**
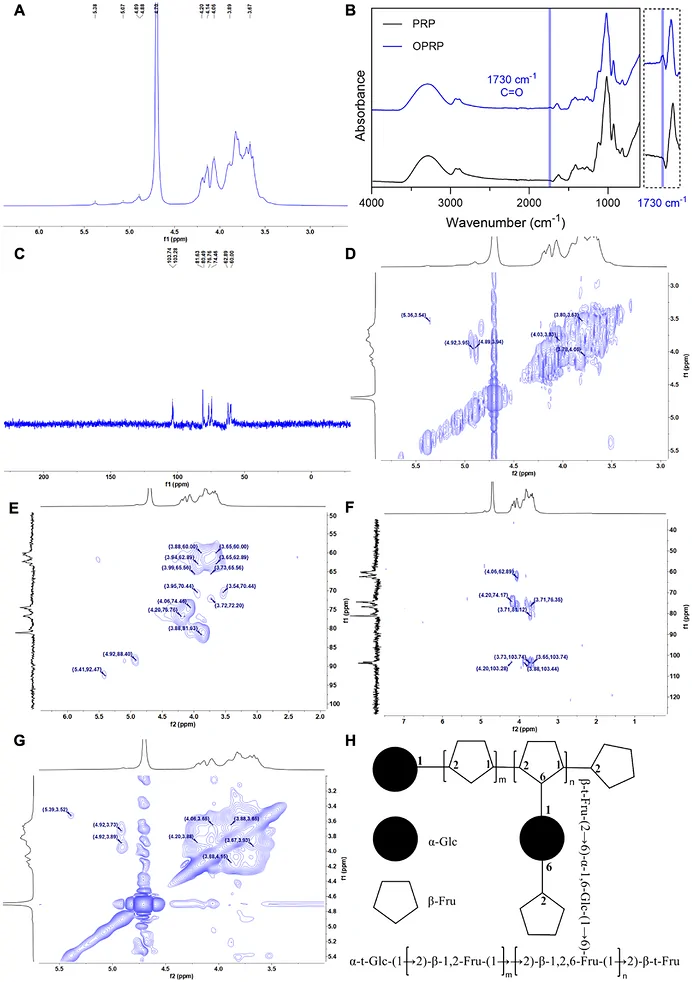
Characterization and structural elucidation of OPRP. (A) ^1^H‐NMR spectrum, (B) the FTIR spectrum, (C) ^13^C NMR spectrum, (D) COSY spectrum, (E) HSQC spectrum, (F) HMBC spectrum, (G) NOESY spectrum, and the proposed structural formula and symbols (H) of OPRP.

The structure of OPRP was further characterized through monosaccharide composition analysis, molecular weight determination, methylation analysis, and multiple NMR analyses. Monosaccharide composition analysis revealed that OPRP consists solely of fructose, glucose, and arabinose, with fructose constituting over 93% (Table S1), preliminarily indicating a neutral inulin‐type structure. Meanwhile, molecular weight determination results showed that the weight‐average molecular weight (Mw) of OPRP is 4.6 kDa, suggesting a low degree of polymerization for this inulin structure. The Mw/Mn ratio of 1.04 indicates low dispersity, confirming the high purity of the isolated polysaccharide. Methylation analysis (Table S2) was employed to determine the glycosidic linkage types in OPRP, revealing five types of residue structures. The most abundant was 1,2‐linked Fru (over 55%), judged to be the main component constituting the backbone. Based on the composition, terminal Glc (t‐Glc) was identified as the initiating glucose residue of the polysaccharide structure, while 1,2,6‐linked Fru was identified as a branched backbone residue. The 1,6‐linked Glc was determined to be part of the side chain, and the 2‐linked Fru was identified as a terminal residue.

The composition and structure of the polysaccharide sample were further confirmed by combining the results from multiple NMR spectra (Figure 2C–G) with the methylation analysis data (Table S2). In the ^1^H NMR spectrum (Figure 2A), within the anomeric region (4.3–5.5 ppm), a small number of anomeric H1 signals from residues could be identified, with the main peaks at 5.38, 5.07, and 4.89 ppm. The number and proportion of protons in the anomeric region were low, consistent with the characteristics of ^1^H NMR spectra for inulin‐type polysaccharides. Concurrently, the C‐H signals in the 3.2–4.3 ppm region correspond to alkyl protons in the residue structure other than anomeric hydrogens, showing numerous and severely overlapping signals, characteristic of polysaccharide ^1^H NMR spectra. In the Figure 2C Nuclear magnetic resonance carbon spectrum (^13^C NMR), no carbonyl carbon signals were observed in the 160–200 ppm range, confirming it is a neutral polysaccharide, which is consistent with both the monosaccharide composition results and the methylation test results for neutral sugars [51]. Within the anomeric region (90–110 ppm), multiple anomeric carbon C1 signals from residues could be identified, mainly at 104.25 and 103.28 ppm, consistent with the carbon spectral features of inulin‐type polysaccharides. Considering the low number of anomeric hydrogens, it is inferred that most signals in the anomeric carbon region correspond to the quaternary C2 carbons of various fructose residues. Multiple carbon signals from residues, excluding anomeric carbons, were present in the 58–85 ppm range. Combined with the downward‐pointing peak in the DEPT spectrum (data not shown), confirming the signals in the 58–64 ppm range are attributed to the ‐CH_2_‐ secondary carbons of fructose methylene C1 and C6.

The polysaccharide structure was further analyzed using two‐dimensional (2D) NMR spectra. In the HSQC two‐dimensional NMR spectrum reflecting hydrogen‐carbon coupling, two weak H1/C1 correlation signals were identified in the anomeric region at 5.41/92.47 and 4.92/88.40 ppm, preliminarily assigned to two types of glucose residues. Notably, the main carbon peaks in the anomeric region had no corresponding proton signals, confirming they belong to C2 signals of fructose, thus validating the assignments of the main carbon signals such as 104.25 and 103.28 ppm. Based on information from this 2D spectrum and combining with reported NMR signals for fructose‐based compounds [52], the major anomeric carbon signals were comprehensively assigned as follows: signals corresponding to a fructan were present, specifically 103.28 ppm for C2 of β‐1,2‐Fru‐(2→, 103.74 ppm for C2 of terminal β‐Fru‐(2→, and 103.94 ppm for C2 of the branched residue β‐1,2,6‐Fru‐(2→. The signal at 5.41/92.47 ppm corresponds to the anomeric H1/C1 of the initiating terminal residue α‐t‐Glc‐(1→, and the signal at 4.92/88.40 ppm corresponds to the anomeric H1/C1 of the residue α‐1,6‐Glc‐(1→ linked to fructose.

By combining signals from multiple 2D NMR spectra, including HSQC, Heteronuclear Multiple Bond Correlation (HMBC), and Correlation Spectroscopy (COSY), the carbon and proton signals of the polysaccharide residues were assigned, as shown in Table S3. Finally, the glycosidic linkage patterns within the polysaccharide structure were determined using HMBC and Nuclear Overhauser Effect Spectroscopy (NOESY) 2D spectra. The proposed chemical structure and schematic of the repeating unit for this inulin‐type fructan, OPRP, are shown in Figure 2H. These findings reveal that OPRP was successfully synthesized and purified. By interpreting various NMR spectra, its structure was identified as an inulin neoseries‐type fructan, and a plausible structural formula for this polysaccharide was proposed.

### 3.2 Preparation, Optimization, and Characterization of COPRP Hydrogel

A bioactive hydrogel COPRP with self‐healing properties was prepared through the Schiff base reaction between the amino group in CMC and the aldehyde group in OPRP. To determine the optimal preparation protocol, various hydrogel formulations and their fundamental properties were summarized in Table S4. Given the limited solubility of the CMC used in this study in deionized water or PBS (maximum solubility of 5 wt.%), this concentration was selected as the final CMC concentration to achieve a hydrogel with a relatively higher modulus and slower degradation rate. As shown in Figure S4 and Table S4, the storage modulus (G’) of the COPRP1 formulation was only about 59 Pa, whereas the G’ values for both the COPRP and COPRP2 formulations exceeded 100 Pa. Present literature demonstrated that relatively soft matrices with stiffness ranging from 0.1 to 1 kPa provide a favorable microenvironment for neural tissue [35, 53]. Consequently, only the COPRP and COPRP2 formulations exhibited sufficient modulus to offer appropriate mechanical support for brain tissue. The porosity of the COPRP hydrogel was approximately 95.4%. Comparative analysis of SEM images and pore size distribution data, i.e., Figure 3G, Figures S2A,B, revealed that the COPRP formulation possessed a moderately sized average pore diameter. Moreover, the pore size distribution of the COPRP hydrogel showed a standard deviation nearly four times smaller than the COPRP2 formulation, indicating superior pore uniformity. Based on the above analysis, the optimized formulation comprising 2.5 wt.% OPRP and 5 wt.% CMC was selected for preparing the smart COPRP hydrogel used in all subsequent characterization and testing. Figure 3A demonstrates successful gelation, showing the transition of the CMC solution to a gel state within 10 min of adding the OPRP solution. The COPRP hydrogel was injected into a heart‐shaped mold using a 30‐gauge (30G) needle with an inner diameter of 0.15 mm. The hydrogel self‐adapted to the shape of the mold shape within 25 min, forming a smooth and intact hydrogel (Figure 3B), which demonstrates the adaptability and self‐healing properties of the COPRP hydrogel at the macroscopic level.

**FIGURE 3:**
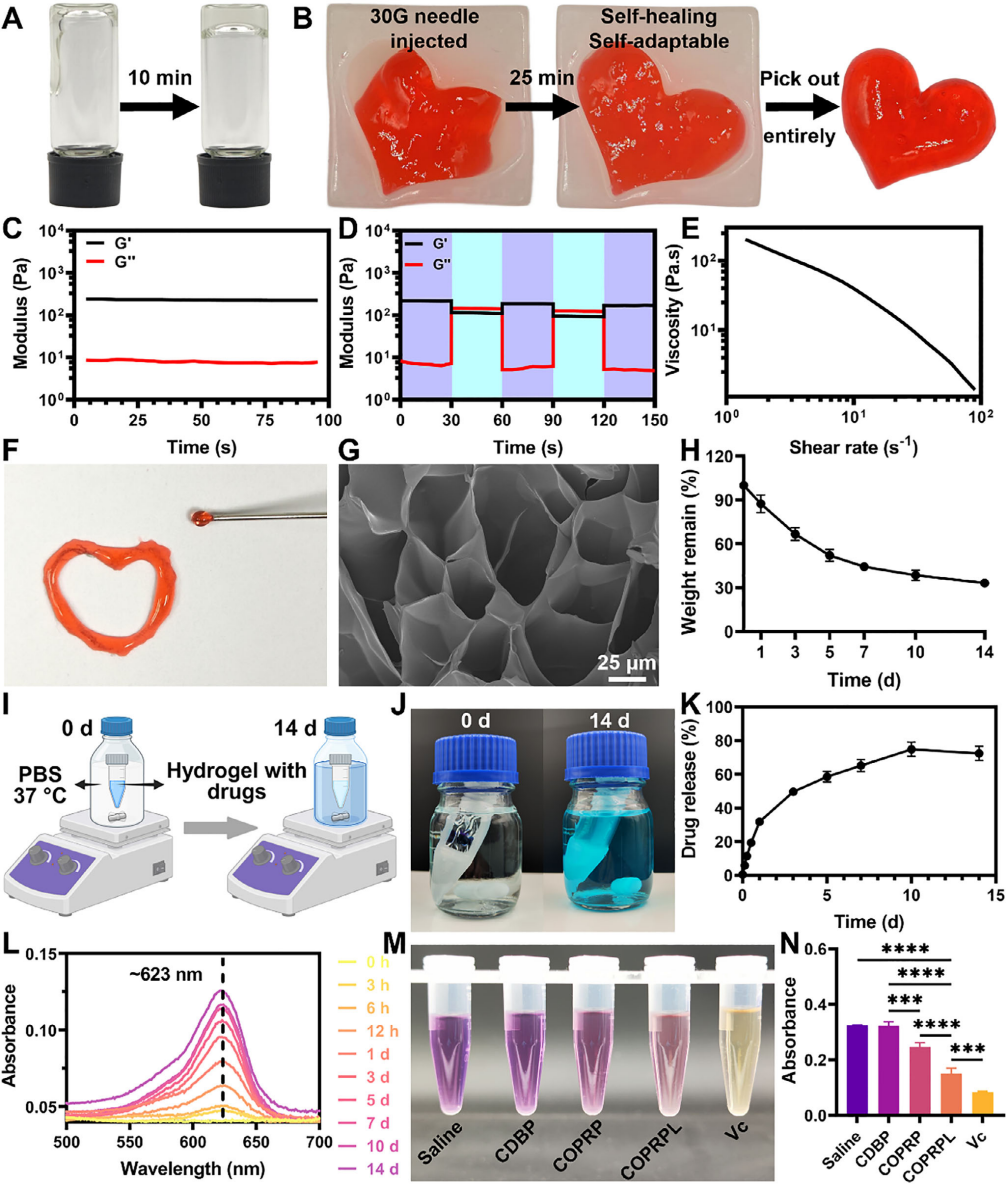
Preparation, morphology, and characterization of the COPRP hydrogel. (A) The macroscopic gelation process of COPRP hydrogel. (B) The COPRP hydrogel can be injected through 30‐gauge syringe needles with a 150 µm internal diameter into the heart‐shaped mold. An integrated heart‐shaped hydrogel with a smooth and homogenous appearance formed after self‐adaption and self‐healing for 25 min at 25°C. (C) Time‐sweep tests of the COPRP hydrogel after complete gelling, showing the steady storage moduli (G′) and loss moduli (G″) of the hydrogel at 1 Hz frequency and 1% dynamic strain. (D) Self‐healing properties of the COPRP hydrogel were investigated through continuous damage–healing cycles at alternate 1 and 380% dynamic strains, respectively. Each step was processed for 30 s at 1 Hz frequency. (E) Shear‐thinning behavior of the COPRP hydrogel was determined by measuring the steady shear viscosity vs. shear rate. (F) The COPRP hydrogel can be injected through 30‐gauge syringe needles to draw the shape of a heart. (G) The SEM image for the cross‐section of the COPRP hydrogel. (H) Weight remaining of the COPRP hydrogel in saline at 37°C. (I) Sustained‐release schematic diagram of COPRP hydrogel loaded with Fast Green FCF (hydrophilic model drug of levodopa), placed in a commercial dialysis tube. (J) Digital photos of COPRP hydrogel in 0 d and 14 d sustained‐release devices. (K) The release profiles calculated by the UV‐vis spectrophotometer. (L) UV‐vis spectra of Fast Green FCF were obtained from the tracer‐containing PBS at different time points. (M) Photos for color changes of DPPH solution after free radical scavenging by various groups of hydrogels, including the negative control (saline), CDBP hydrogel, COPRP hydrogel, COPRPL hydrogel, and the positive control (ascorbic acid). (N) The absorbance of DPPH solution in each group was detected via UV‐vis. All data are represented as mean ± STD (n ≥ 3). ^*^ *p* < 0.05, ^**^ *p* < 0.01, ^***^ *p* < 0.001, and ^****^ *p* < 0.0001 between the indicated groups.

The mechanical properties of the COPRP hydrogel were evaluated microscopically using rheometry. A time‐sweep experiment conducted at 1 Hz frequency and 1% strain showed a stable G′ of approximately 230 Pa for COPRP hydrogel, as shown in Figure 3C. Amplitude sweep test (Figure S3) determined the critical strain for the transition from a gel state (G′ > G″) to a sol state (G′ < G″) to be 370%, as the dynamic strain increased from 1% to 560%. Therefore, damage‐healing cycles, i.e., rheological self‐healing test, were performed by alternating between 380% (high) and 1% (low) strain. The hydrogel reversibly transitioned to a sol state under high strain and recovered its gel state with a modulus nearly identical to the original upon returning to low strain (Figure 3D), demonstrating proper self‐healing behavior at the microscopic level. Steady shear rate tests revealed a smooth decrease in viscosity with increasing shear rate in Figure 3E, confirming the shear‐thinning behavior and injectability of the hydrogel. This property was further corroborated macroscopically by the smooth extrusion and writing of letters through a 30G needle for the hydrogel, which is performed in Figure 3F. SEM image in Figure 3G revealed a uniform porous structure in the cross‐section of the COPRP hydrogel, with an average pore size of approximately 45.4 µm. Furthermore, the hydrogel retained about 34% of its initial mass after 14 days of degradation in saline, with a slightly decelerated degradation rate, supporting the selection of a 14‐day duration for in vivo rat models (Figure 3H). However, the actual degradation window in vivo will be considerably shortened due to factors such as body fluids, inflammatory environments, and cellular infiltration.

A schematic of the sustained release experiment is shown in Figure 3I. The COPRP hydrogel loaded with a hydrophilic model drug, i.e., FCF, was placed in a dialysis device under stirring at 37°C for 14 days. Since the molecular weight of FCF is lower than the retention threshold of the dialysis membrane, the drug diffuses into the surrounding PBS buffer. Corresponding digital images displayed in Figure 3J showed the buffer turning from colorless to deep blue over 14 days, while the color of the gel inside the device faded, visually confirming sustained release. The cumulative release profile, calculated from UV‐vis spectroscopy data (Figure 3K,L), showed steady release over 14 days, reaching a plateau starting from about 10 days to the end point. These findings demonstrate the sustained release of a hydrophilic model compound from COPRP and support its feasibility for drug loading, although the release kinetics of levodopa itself require direct characterization.

Free radical scavenging assays, demonstrated in Figure 3M,N for both the blank hydrogel (CDBP) and the negative control (Saline) groups, exhibited a deep purple color with no significant difference in absorbance at 517 nm. In contrast, the COPRP hydrogel and COPRPL hydrogel groups possessed lighter purple hues. The COPRPL hydrogel group, in particular, showed the lightest color among experimental groups, with significantly lower absorbance than the Saline and CDBP hydrogel groups, though still higher than the positive control (Vc) group. These results indicate that the incorporation of OPRP imparts free radical scavenging properties to the hydrogel, likely inheriting this characteristic from the parent PRP. Studies have reported that PRP exhibits potent antioxidant activity against free radicals, lipid peroxidation, and protein glycation [54, 55]. In addition, the COPRPL hydrogel group exhibited an over 2‐fold enhancement in scavenging capacity compared to the COPRP group, which is attributed to the strong antioxidant capacity on a chemical level conferred by the polyphenolic groups of levodopa [56, 57, 58]. This additional antioxidant effect may serve as a foundation for subsequent development of the COPRP bioactive hydrogel platform for loading various clinical drugs.

### 3.3 In Vitro Antioxidant and Anti‐Inflammatory Capacities

All in vitro cell experiments were conducted in Transwell chambers within 24‐well plates. First, SH‐SY5Y cells were cultured with different concentrations of levodopa to screen for the optimal administration concentration via the CCK‐8 assay in Figure S5. On day 2, the 25 µm levodopa treated group showed the highest cell viability with about 400% proliferation, which has a statistically significant difference compared to other groups. Therefore, 25 µm levodopa was selected for the drug‐loading concentration in COPRPL hydrogel in subsequent experiments. To evaluate the antioxidant properties of the COPRP hydrogel in vitro, BV2 cells were stimulated with LPS to generate ROS. The ability of each hydrogel group to scavenge ROS was assessed using an ROS detection kit, as shown in Figure 4A, with the results of average fluorescence intensity quantification presented in Figure S6. Compared to the positive control group with saline, the average fluorescence intensity in the CDBP hydrogel group was slightly lower, but there was no significant difference between the two groups. The CMC in the CDBP hydrogel likely primarily affects its water solubility and biocompatibility, and although CMC possesses some antioxidant activity, such activity is insufficient to produce a significant ROS‐scavenging effect. However, hydrogel groups containing OPRP showed significant differences compared to both the Saline and CDBP hydrogel groups, effectively clearing ROS. Concurrently, multiple studies have also indicated that inulin‐type fructans can effectively mitigate oxidative stress in disease models [59, 60, 61], highlighting the potential of OPRP as an antioxidant. Among the LPS‐challenged groups, COPRPL exhibited the lowest ROS fluorescence, whereas drug‐free COPRP also produced a substantial antioxidant effect. Because the present experiment was not designed to quantify pharmacological interactions, these findings should not be interpreted as evidence of formal synergy. Rather, they demonstrate that OPRP retains its antioxidant activity after hydrogel formation and that levodopa loading provides an additional reduction in cellular ROS. The rationale for COPRPL is therefore based on complementary functions: COPRP actively modulates the oxidative and inflammatory microenvironment, whereas levodopa provides dopamine‐precursor supplementation.

**FIGURE 4:**
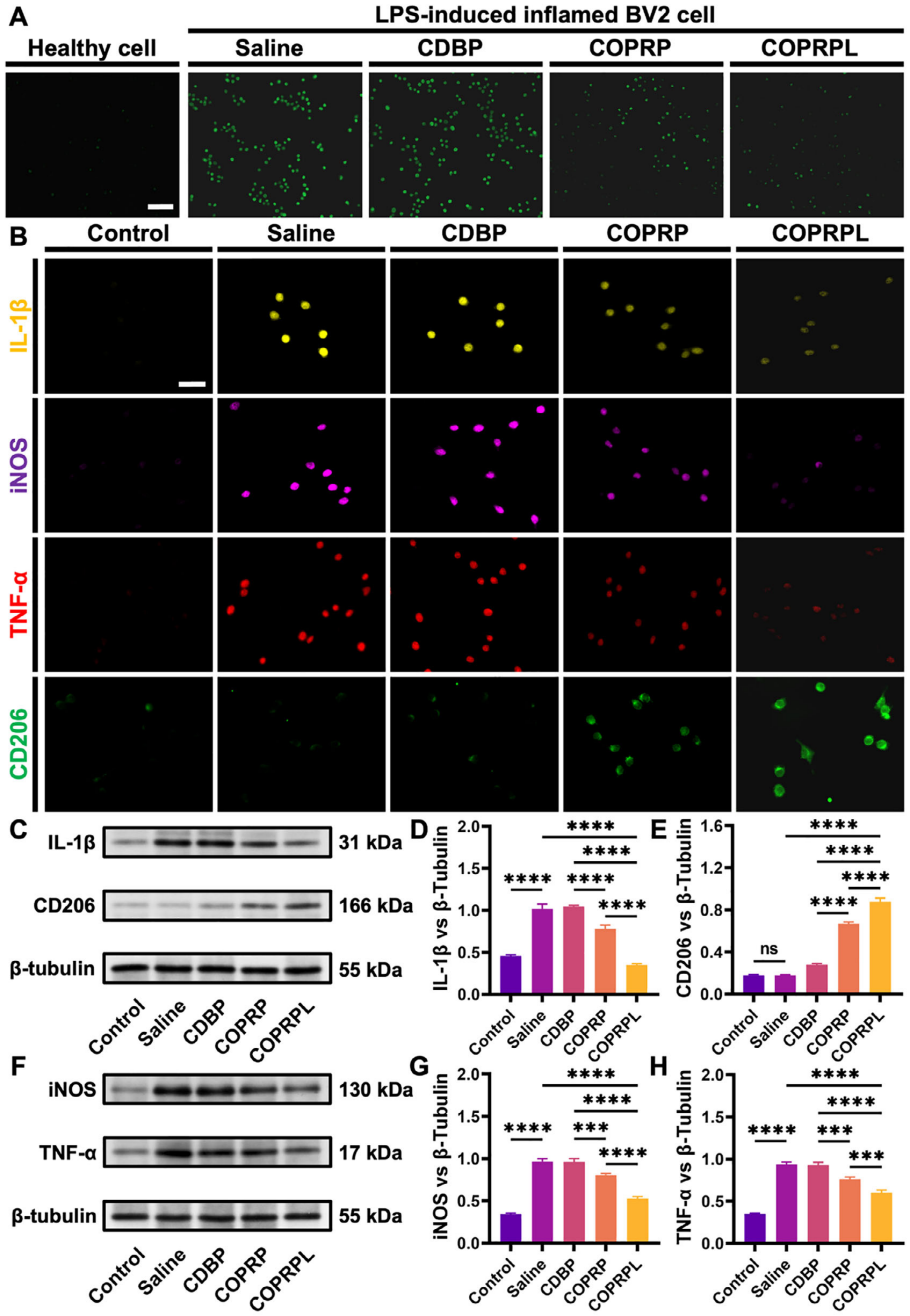
In vitro cellular reactive oxygen species (ROS) scavenging and anti‐inflammatory experiments. (A) IF images for ROS of LPS‐induced BV2 microglia and each group treatment by DCFH‐DA staining. The scale represents 100 µm. For anti‐inflammatory capacity, BV2 cells were pre‐induced by LPS toward the inflamed M1 phenotype. (B) IF images of IL‐1β, iNOS, TNF‐α, and CD206 in LPS‐induced BV2 cells after 12 h of treatment. The scale represents 50 µm. (C) Western blot (WB) analysis validated the expression of IL‐1β and CD206. Densitometric analysis of (D) IL‐1β and (E) CD206 protein expression, normalized against β‐tubulin expression. (F) WB analysis for other inflammatory factors, i.e., TNF‐α and iNOS. Densitometric analysis of (G) iNOS and (H) TNF‐α protein expression, normalized against β‐tubulin expression. All data are represented as mean ± STD (n ≥ 3). ^*^ *p* < 0.05, ^**^ *p* < 0.01, ^***^ *p* < 0.001, and ^****^ *p* < 0.0001 between the indicated groups.

The anti‐inflammatory capacity of the COPRP hydrogel against LPS‐induced inflammation in BV2 cells was investigated via IF staining (Figure 4B) and WB analysis (Figure 4C,F), focusing on M1‐type markers (IL‐1β, iNOS, TNF‐α) and the M2‐type marker (CD206). The uncropped images for WB in Figure 4 are presented in Figures S7 and S8. The average fluorescence intensities from the IF experiment were quantified in Figure S9, while the statistical results of the WB bands are displayed in Figure 4D,E,G,H. The IF results showed that compared to the Saline group (treated with LPS only), the COPRP hydrogel groups significantly reduced the average fluorescence intensity of the three M1‐type pro‐inflammatory markers (with the reduction in IL‐1β being most pronounced). The COPRP hydrogel group showed a reduction of > 40%, and the COPRPL hydrogel group showed a reduction of > 50%. Conversely, the COPRP hydrogel group significantly upregulated the expression of the M2‐type anti‐inflammatory factor CD206 (over 1.8‐fold increase), and the COPRPL hydrogel group exhibited an over 3‐fold upregulation. The WB results were consistent with the IF findings. Previous research has demonstrated that polysaccharides are one of the main active components of *Polygonati rhizoma*, possessing properties such as anti‐inflammatory and antioxidant activities. This study also confirmed that hydrogels containing bioactive OPRP exhibit significant anti‐inflammatory effects, effectively alleviating cellular inflammation in vitro.

### 3.4 Efficacy Evaluation of In Vitro Cellular PD Model

6‐OHDA‐induced SH‐SY5Y cells, a human neuroblastoma cell line, were established as a reliable in vitro cellular PD model for the present study upon modification of a preexisting procedure [62, 63, 64]. SH‐SY5Y cells basally express key markers of dopaminergic neurons for neurodegenerative diseases, such as TH and dopamine transporter, and are considered to closely resemble immature catecholaminergic neurons [62, 65]. Furthermore, 6‐OHDA is structurally similar to endogenous dopamine. It is a highly reactive and readily auto‐oxidizable catecholamine analog that can be specifically taken up by SH‐SY5Y cells via the dopamine transporter, thereby mimicking the key feature of selective dopaminergic neuron damage in PD. After entering the cells, 6‐OHDA undergoes auto‐oxidation and inhibits the mitochondrial electron transport chain, triggering a burst of ROS accumulation and leading to severe oxidative stress. This intense oxidative damage results in decreased cell viability. Concurrently, oxidative stress can induce the opening of the mitochondrial permeability transition pore, directly causing a decrease in the mitochondrial membrane potential (MMP). Moreover, MMP depolarization is associated with neuronal injury and reduced TH expression. It also promotes cytochrome c release and caspase activation, ultimately leading to apoptosis [66, 67]. We therefore evaluated cell viability, apoptosis, MMP, and TH expression to assess COPRP‐mediated protection in the 6‐OHDA model.

Cell viability, i.e., 6‐OHDA‐induced and rescue assay, was measured using the CCK‐8 kit. As shown in Figure S10, after SH‐SY5Y cells were induced with 100 µm 6‐OHDA for 24 h (Day 0), the cell viability of the treated group decreased to 80% of the initial state, confirming that 6‐OHDA successfully induced a reliable cytotoxic model. After three days of treatment with the respective experimental groups, the cell viability in the Saline group (saline treated after 6‐OHDA‐induced) and the CDBP hydrogel group showed no significant recovery over the three days, with no statistical difference between them (*p* > 0.05), indicating that neither the injury environment alone nor the blank bioinert hydrogel itself could effectively promote the recovery of cell viability. In contrast, the COPRP hydrogel group (containing OPRP active components) exhibited a significant protective effect, with cell viability significantly increased compared to the Saline and CDBP hydrogel groups (*p* < 0.01). The COPRPL hydrogel group (COPRP hydrogel loaded with levodopa) demonstrated the best therapeutic efficacy among all treatment groups, with its degree of cell viability recovery being moderately higher than that of the COPRP hydrogel group (99.17% vs. 112.1%, *p* < 0.05). The results indicate that OPRP‐containing bioactive hydrogel can effectively counteract the cytotoxicity induced by 6‐OHDA, while also enhancing efficacy when loaded with a clinical drug, thereby promoting cell survival and proliferation.

Cell apoptosis experiment was detected using FITC‐labeled Annexin V and PI to distinguish between early apoptosis and necrosis/late apoptosis, as demonstrated in Figure 5A. Quantitative analysis of their average fluorescence intensity was performed in Figure 5B,C. No fluorescence was detected in the healthy cells of the Control group, while the Saline group exhibited strong dual fluorescence, indicating that 6‐OHDA successfully induced apoptosis in SH‐SY5Y cells. The CDBP hydrogel group also showed strong dual fluorescence, with almost no difference from the Saline group. However, the fluorescence intensities of both Annexin V‐FITC and PI were significantly reduced in the COPRP hydrogel and COPRPL hydrogel groups (*p* < 0.05), and the numbers of cells in early apoptosis and necrosis/late apoptosis were both significantly decreased. The results indicate that the COPRP hydrogel can significantly alleviate 6‐OHDA‐induced cell apoptosis, and the COPRP hydrogel containing levodopa showed a more pronounced effect.

**FIGURE 5:**
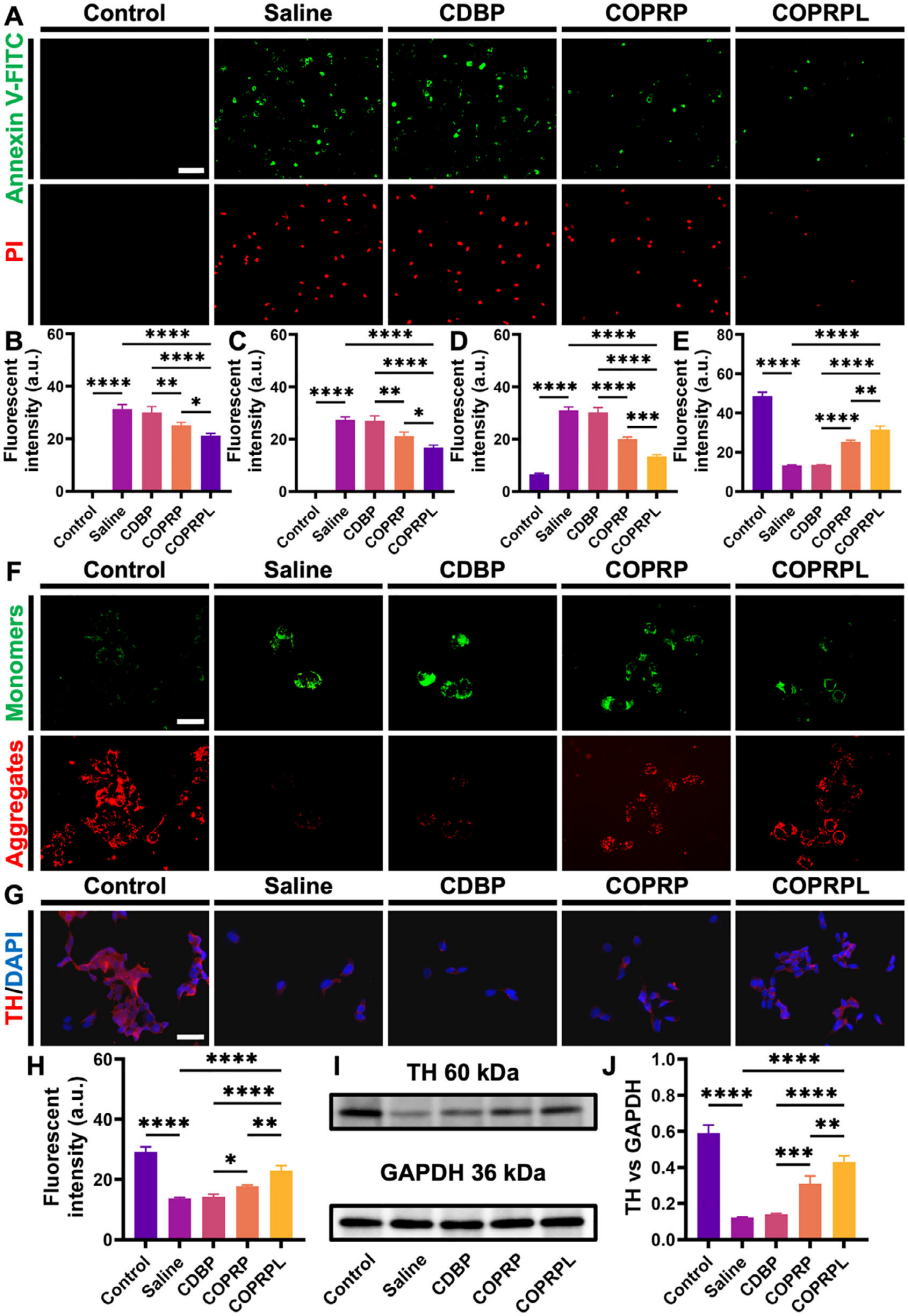
In vitro cellular PD model experiments. SH‐SY5Y cells were treated with 6‐hydroxydopamine (6‐OHDA) for 24 h to establish an in vitro cell PD model. (A) Fluorescence images of apoptosis in 6‐OHDA‐induced SH‐SY5Y cells co‐cultured with each experimental group for 24 h were detected using Annexin V‐FITC/PI staining. The scale represents for 100 µm. The mean fluorescence intensities of (B) Annexin V‐FITC and (C) PI in each group were quantified and presented in graphs. (F) Fluorescence microscopy images of mitochondrial membrane potential in 6‐OHDA‐induced SH‐SY5Y cells after 24 h of treatment with each experimental group. The scale represents 50 µm. The average fluorescence intensities of (D) monomers and (E) aggregates in each group were quantified and presented in graphs. After a 24‐h co‐culture between 6‐OHDA‐induced SH‐SY5Y cells and each experimental group, (G) IF images of tyrosine hydroxylase (TH)‐positive cells, (H) quantified average fluorescence intensity results, and (I) TH protein expression by WB. The scale represents 50 µm. TH protein expression intensity was normalized against GAPDH for optical density analysis (J). All data are represented as mean ± STD (n ≥ 3). ^*^ *p* < 0.05, ^**^ *p* < 0.01, ^***^ *p* < 0.001, and ^****^ *p* < 0.0001 between the indicated groups.

The JC‐1 dye selectively entered the mitochondria in the MMP assay (Figure 5F). As the membrane potential decreases, the color can reversibly change from red to green. The corresponding average fluorescence intensities for each experimental group are presented in Figure 5D,E. The results show that healthy Control group cells had high MMP, with JC‐1 forming aggregates producing strong red fluorescence. The Saline and CDBP hydrogel groups exhibited low MMP, with JC‐1 remaining in monomer form, producing almost exclusively green fluorescence. Compared to the Saline and CDBP hydrogel groups, both the COPRP hydrogel and COPRPL hydrogel groups significantly enhanced the MMP, with significantly increased red fluorescence from aggregates (*p* < 0.0001) and significantly decreased green fluorescence from monomers (*p* < 0.0001). This indicates that the COPRP hydrogel containing OPRP can effectively increase the MMP in 6‐OHDA‐induced SH‐SY5Y cells, regulating mitochondrial function.

To evaluate the protective effect of different treatments on TH, the expression level of TH in SH‐SY5Y cells from different experimental groups was detected by IF in Figure 5G, and the fluorescence intensity was quantitatively analyzed in Figure 5H. The uncropped images for WB in Figure 5 are presented in Figure S11. The results show that the Control group had the highest TH fluorescence intensity with the fluorescence intensity ∼1.7‐fold higher than that of the saline group (*p* < 0.0001), indicating that 6‐OHDA successfully induced damage in the dopaminergic neuron model, leading to downregulation or loss of TH protein expression. Compared to the Saline group, the CDBP hydrogel group showed no significant difference in TH fluorescence intensity, suggesting that the CMC‐based bioinert hydrogel itself does not possess a protective effect against 6‐OHDA‐induced TH downregulation. However, the fluorescence intensity of TH in the COPRP hydrogel group showed a significant upregulation (∼1.15‐fold) compared to the Saline and CDBP hydrogel groups (*p* < 0.05), indicating that the OPRP active components can effectively against the toxicity of 6‐OHDA, partially maintaining TH expression. The COPRPL hydrogel group exhibited the strongest protective effect, with TH fluorescence intensity being the highest among all treatment groups (∼1.9‐fold higher than the Saline group) and also ∼1.19‐fold higher than that of the COPRP hydrogel group (*p* < 0.01). Furthermore, Figure 5I shows the protein expression levels of TH in SH‐SY5Y cells from different experimental groups via WB analysis. Figure 5J shows the quantitative statistical results of the WB bands, which are consistent with the trends observed in the TH IF staining results: no significant difference between the CDBP group and the Saline group. Compared with the Saline group and the CDBP hydrogel group, the COPRP and COPRPL hydrogel groups containing OPRP active agents exhibited a significant increase in TH protein expression, with the COPRPL hydrogel group demonstrating the greatest protective effect (0.3116 vs. 0.4304, *p* < 0.05). The loading of levodopa was evident in recovering TH function in the cellular PD model, though still not multiplicative, which may correlate with its excellent clinical efficacy in PD [34], with in vivo effects subsequently validated.

In vitro cellular PD model indicates that the CDBP hydrogel group showed no significant improvement in any of the above experimental indicators. COPRP hydrogels containing OPRP active components exhibit multidimensional protective effects by scavenging ROS and alleviating inflammation: significantly restoring cell viability, enhancing MMP, alleviating cell apoptosis, and upregulating TH expression. Notably, COPRPL provided additional protection relative to COPRP in these assays, supporting complementary effects of OPRP‐mediated microenvironmental modulation and levodopa supplementation rather than formal synergy. The above experimental results suggest that the bioactive COPRP hydrogel may serve as a dual‐function bioactive platform with therapeutic effects for further application in neuroprotective therapy for PD.

### 3.5 Behavioral Evaluation of Rat PD Model

The therapeutic potential of the COPRP hydrogel was evaluated in vivo using an SD rat model of PD with 6‐OHDA‐induced unilateral mfb lesions to assess therapeutic efficacy and behavioral outcomes. The 6‐OHDA‐induced PD rat model provides a stable simulation of the progressive degeneration of the nigrostriatal pathway and PD behavioral symptoms [68]. As a classic model for evaluating neuroprotective interventions, the model closely mimics the progressive course of neurodegeneration in human PD [69]. On days 7 and 14 post‐treatment, the recovery of motor function in PD rats following drug‐loaded hydrogel treatment was systematically evaluated using the circling test, cylinder test, and open‐field test, which is systematically summarized in Figure 6A.

**FIGURE 6:**
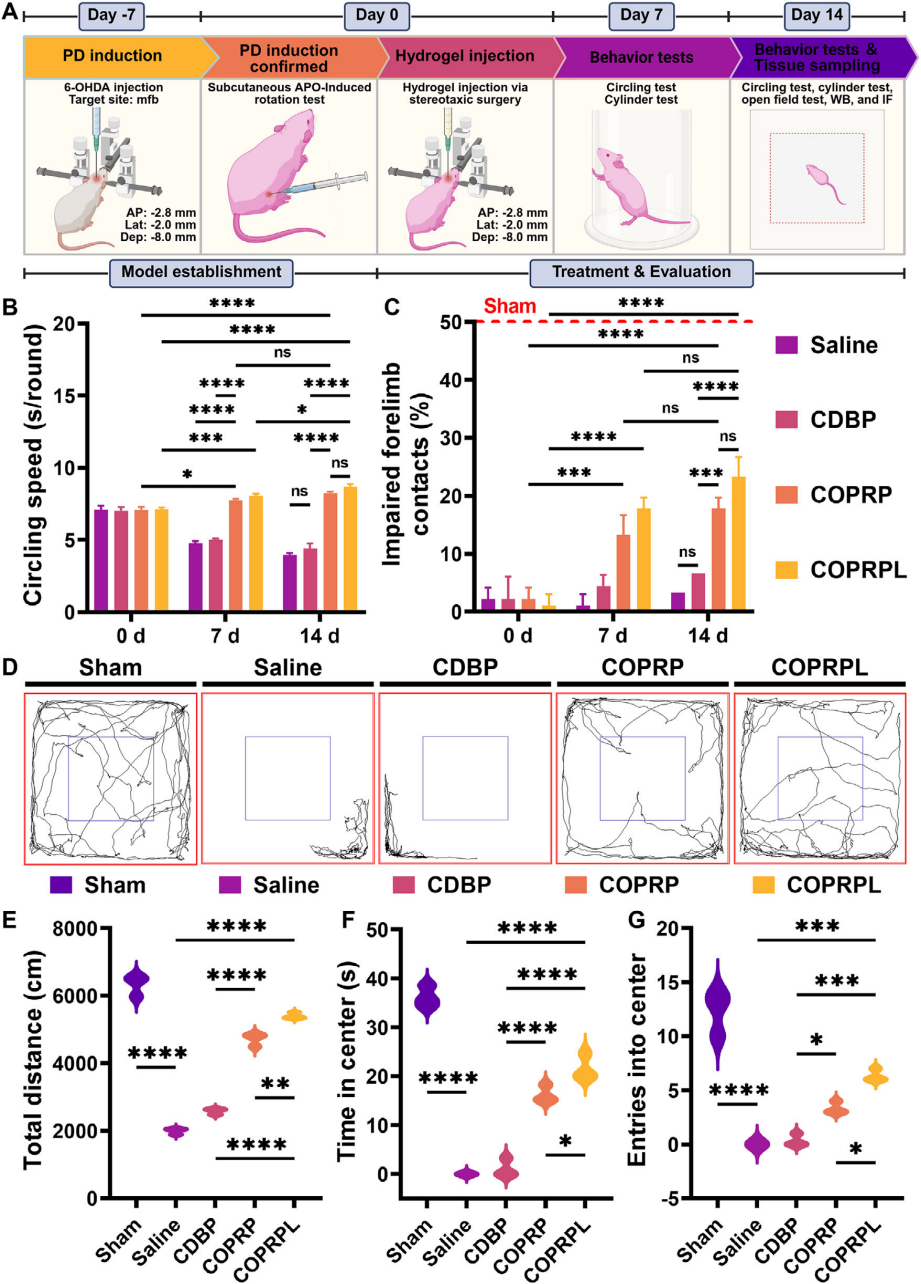
Behavioral evaluation of PD rats treated by hydrogels. (A) Schematic illustration of the PD rat model. The PD model was established by in situ injection of 6‐OHDA into the right mfb region of rat brains. After confirmation of PD induction, the hydrogels were further injected into the lesion regions for 14 days, and the efficacy of treatment was evaluated through behavior tests, IF analyses, and WB analyses. Images created with BioRender.com. Functional recovery in each group of PD rats was quantitatively assessed based on (B) the rotational asymmetry speeds and (C) the impaired forelimb contact proportion. (D) Total distance traveled and line crossing in the open field with the center zone. (E) Total traveled distance, (F) time spent in the center zone, and (G) entries into the center zone were counted through Smart 3.0 software. All data are represented as mean ± STD (n ≥ 3). ^*^ *p* < 0.05, ^**^ *p* < 0.01, ^***^ *p* < 0.001, and ^****^ *p* < 0.0001 between the indicated groups.

APO‐induced rotational behavior is one of the most used criteria for assessing the severity of unilateral nigrostriatal lesions and the response to treatment [69, 70]. APO induces rats to turn toward the unaffected side by activating D2 receptors on the lesioned side, which have become hypersensitive due to denervation, and a reduction in turning rate typically reflects partial recovery of dopaminergic function on the lesioned side [36, 71]. As shown in Figure 6B, the circling speed in the Saline group remained consistently high (<4.9 s/round) throughout the 14‐day observation period, indicating that the 6‐OHDA‐induced lesion was stable and showed no spontaneous recovery. The rotational behavior in the CDBP hydrogel group was not significantly different from the Saline group. Notably, the rotation time in the COPRP hydrogel group was already significantly increased than those in the Saline and CDBP hydrogel groups by day 7 (*p* < 0.0001) and rotation rate further decreased by day 14 (∼8.2 s/round), confirming that the oxidatively modified and crosslinked COPRP preserved the neuroprotective activity in vivo. The COPRPL hydrogel group demonstrated optimal efficacy as early as day 7 of treatment, with the circling speed dropping to the lowest level among all treatment groups. Findings provide functional evidence that levodopa is continuously released from the hydrogel, exerting a supplementary dopaminergic effect locally within the brain, thereby improving motor symptoms. The cylinder test serves to evaluate asymmetry in forelimb use with high sensitivity for spontaneous forelimb use deficits in rodents [72, 73]. Healthy rats use both forelimbs equally to contact the cylinder wall during upright exploration, whereas unilateral 6‐OHDA injury leads to a significant reduction in the use of the contralateral forelimb. As depicted in Figure 6C, the contralateral forelimb usage rate in rats on day 0 was only approximately 2%, indicating significant spontaneous movement asymmetry. After therapy for 14 days, all groups treated with OPRP‐crosslinked hydrogels showed a significant improvement in contact rate of the damaged forelimb compared to the saline group, except for the CDBP inert hydrogel group. Each group demonstrated over 15% recovery in forelimb contact, implying that OPRP can partially alleviate motor asymmetry. The COPRPL hydrogel group containing levodopa achieved the most significant improvement among all treatment groups, showing more than 22% recovery in forelimb contact, hinting that continuous supplementation with levodopa further promoted the recovery of forelimb motor function deficits. However, in the 14‐day circling and cylinder tests, no statistically significant difference was observed between the COPRP and COPRPL groups, perhaps indicating that the therapeutic effects of these two behavioral measures have reached an upper limit or are constrained by the dosage administered.

Open‐field test was applied to evaluate spontaneous locomotion and exploratory behavior in rats in an unfamiliar environment (Figure 6D–G) [74]. Both the total distance traveled and the time remaining in the central area were significantly lower in the Saline group and the CDBP inert hydrogel group compared to the Sham group (*p* < 0.0001), reflecting the motor bradykinesia and reduced exploratory motivation typical of PD. In contrast to the Saline group, the total distance traveled, time spent in the central area, and number of entries were all increased in the OPRP‐containing hydrogel‐treated groups, particularly in the COPRPL group, with statistically significant differences. Taken together, the results of the three behavioral assessments showed a clear gradient of improvement in therapeutic efficacy across the various hydrogel‐treated groups. Notably, the CDBP hydrogel group, being bioinert, failed to effectively improve behavioral deficits in PD rats across all behavioral tests. Conversely, the OPRP‐crosslinked COPRPL hydrogel revealed statistically significant behavioral recovery in the rotational speed test, the cylinder asymmetry test, and the open field test. Earlier literature has reported that PRP can reduce dopaminergic neuron apoptosis by inhibiting oxidative stress [75] while also regulating neuroinflammation by suppressing excessive microglial activation and modulating M1/M2 polarization [16]. These effects may collectively contribute to the behavioral improvements observed in PD rats treated with COPRP hydrogels, suggesting that COPRP hydrogels may possess multidimensional capabilities in vivo, including anti‐apoptotic, antioxidant, and neuroinflammatory regulatory effects. Furthermore, Li et al. [75] and Zhang et al. [76] reported that PRP effectively improves motor deficits in PD mouse models, supporting the efficacy of COPRP hydrogels in restoring behavioral symptoms in PD models. Levodopa loading was included as a proof‐of‐concept to demonstrate the compatibility of COPRP with a clinically relevant PD drug, and COPRPL produced additional improvements in several behavioral outcomes. These findings therefore support COPRP as an intrinsically bioactive matrix capable of drug loading, while its delivery‐specific benefits require further comparative investigation.

### 3.6 Neuroprotective Effects and Regulation of Neuroinflammation

Brain tissue sections were first analyzed using H&E staining (Figure S12). At 7 days post‐injection, the hydrogel was observed to have degraded almost completely in the coronal sections, with only a few traces and a small number of immune cells remaining. By 14 days post‐injection, the number of immune cells in the coronal sections had returned to normal levels. The in vivo degradation rate was slightly faster than the in vitro results, which may be related to the inflammatory environment and cellular infiltration. To preliminarily evaluate systemic histocompatibility, H&E staining of the heart, liver, spleen, lung, and kidney was performed at the experimental endpoint. No apparent treatment‐related histopathological abnormalities were observed in any of the examined organs across the experimental groups (Figure S13). These histological findings are preliminary because blood biochemical indices were not assessed. Despite multiple lines of indirect evidence supporting the favorable local tolerability of COPRP in the brain, the absence of COPRP‐treated sham‐operated healthy animals precludes a definitive assessment of its biological effects in the intact brain and its long‐term safety. IF staining of coronal brain sections from PD rats revealed significant changes in TH, a characteristic marker of PD, following hydrogel treatment. Figure 7A showed fluorescence images of TH‐positive dopaminergic neurons in the substantia nigra compacta (SNc), and Figure 7B summarized the semi‐quantitative data. Compared with the negative control group treated with saline, the number and fluorescence intensity of TH‐positive dopaminergic neurons in the SNc of PD rats treated with the biomaterial were significantly increased, particularly in the group treated with OPRP‐crosslinked bioactive hydrogels. Among these two groups, the TH fluorescence intensity in the levodopa‐containing COPRPL hydrogel group also increased by approximately 1.2‐fold compared to the COPRP hydrogel group, which was consistent with the results of TH intensity in in vitro cellular PD model.

**FIGURE 7:**
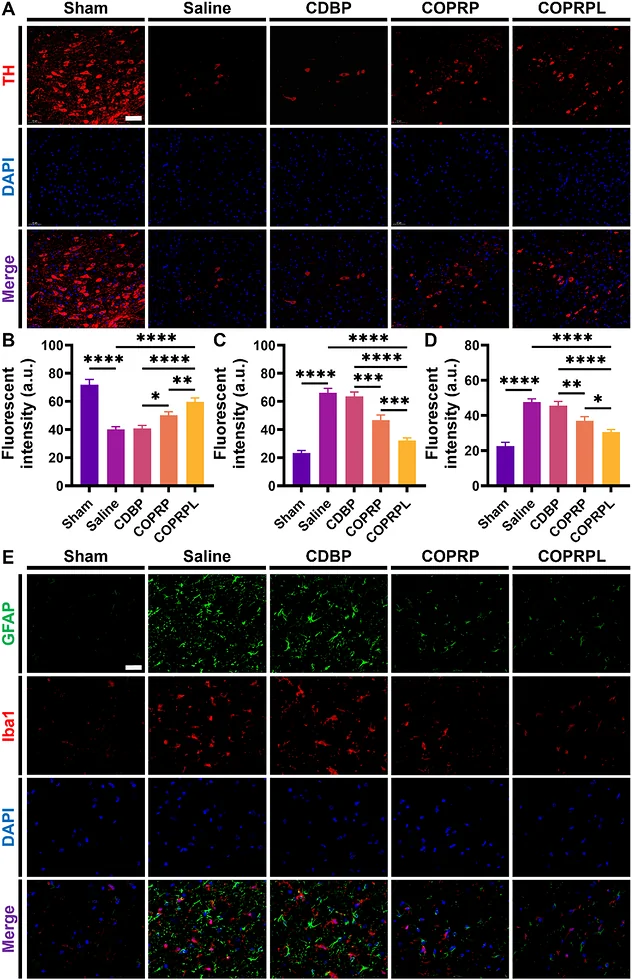
In vivo histological analysis of PD‐specific markers and neuroinflammation in the substantia nigra (SNc). (A) Expression of TH‐positive dopaminergic neurons in the SNc was assessed 14 days after the second surgical injection. (B) The mean fluorescence intensity of TH‐positive dopaminergic neurons in the SNc across groups was quantified and presented graphically. (E) Fluorescence images of GFAP‐positive astrocytes (green) and Iba1‐positive microglia (red) 14 days after implantation. Quantitative results of in vivo fluorescence intensity for (C) GFAP‐positive astrocytes and (D) Iba1‐positive microglia. Both scale bars represent 40 µm. All data are represented as mean ± STD (n ≥ 3). ^*^ *p* < 0.05, ^**^ *p* < 0.01, ^***^ *p* < 0.001, and ^****^ *p* < 0.0001 between the indicated groups.

The microglial marker Iba1 and the astrocytic marker GFAP were further evaluated by IF staining, and the representative images are presented in Figure 7E. Semi‐quantitative analysis shown in Figure 7C,D revealed that, as PD pathology progressed, the numbers of activated microglia (Iba1‐positive) and GFAP‐positive astrocytes in the Saline group were significantly greater than those in all other groups. In contrast, both the COPRP‐ and COPRPL‐treated groups exhibited marked reductions in Iba1‐positive microglia and GFAP‐positive astrocytes, indicating that these two treatments substantially attenuated neuroinflammation in the SNc region. Notably, the COPRPL hydrogel‐treated group displayed elevated Iba1‐positive microglial levels relative to the Sham group, whereas GFAP‐positive astrocyte levels were only marginally upregulated. As reported in previous studies, excessive activation of GFAP‐positive astrocytes can exacerbate PD by generating an acidic, pro‐inflammatory microenvironment that promotes microglial activation and perpetuates neuroinflammatory cascades, whereas a moderate astrocytic response promotes neuroprotection through the release of neurotrophic growth factors [38]. Furthermore, one of the primary mechanisms by which 6‐OHDA induces PD involves the impairment of neuronal mitochondrial function and the subsequent overproduction of ROS [77]. The antioxidant capacity at the tissue level across groups was assessed by measuring SOD activity, as shown in Figure S14. Among all treatment groups, the COPRPL hydrogel‐treated group exhibited the highest cerebral SOD activity (approximately 53 U/mg tissue), which was ∼1.15‐fold higher than that of the COPRP hydrogel‐treated group, yet remained ∼1.20‐fold lower than that of the Sham group. Collectively, these results confirm that OPRP bioactivity is preserved in vivo and that levodopa loading provides an additional therapeutic effect. Previous studies have rarely explored the mechanisms of PRP treatment in PD, though some research exists regarding spinal cord injury repair and kidney disease treatment. PRP can inhibit microglial activation in spinal cord injury, but this is achieved by first influencing the gut microbiota, followed by regulatory effects [44]. Additionally, PRP treatment for diabetic kidney disease in mice has demonstrably achieved therapeutic effects by targeting the TGF‐β/Smad2 signaling pathway and possessing pronounced in vivo antioxidant properties [78].

### 3.7 Proteomics Analysis and Targeting TGF‐β/Smad Pathway for PD Treatment

Because COPRP improved therapeutic outcomes in the rat PD model, we compared the brain proteomes of CDBP‐ and COPRP‐treated rats. Principal Component Analysis (PCA) clearly separated the two groups, indicating differences in their global protein‐abundance profiles (Figure 8A). Differential expression analysis identified a significant set of proteins uniquely regulated by the COPRP treatment (Figure 8B). Among these, 161 proteins were up‐regulated, while 38 proteins were down‐regulated. The heatmap illustrated coordinated expression changes within a relevant gene set (Figure 8C). Subsequently, we performed Kyoto Encyclopedia of Genes and Genomes (KEGG) pathway enrichment analysis on these differentially expressed proteins (DEPs), revealing that DEPs are closely associated with focal adhesion, the sphingolipid signaling pathway, axon guidance, the Rap1 signaling pathway, and the neurotrophins signaling pathway (Figure 8D). Focal adhesion, multi‐protein complexes that link the extracellular matrix to the intracellular cytoskeleton, are key mediators of cell adhesion, migration, and proliferation [79]. They play a complex and crucial role in PD, primarily involving the regulation of neuronal survival, synaptic function, and neuroinflammation [80, 81]. Among the focal‐adhesion‐related DEPs, Col6a1, Col6a2, Col6a3, Pxn, and Tln1 were upregulated, whereas Raf1 and Itgb1 were downregulated (Figure 8E). This pattern suggests selective remodeling of extracellular‐matrix and adhesion‐related signaling rather than uniform activation of the entire pathway. This suggests that COPRP treatment activates and enhances adhesion signaling between cells and the extracellular matrix within the brain.

**FIGURE 8:**
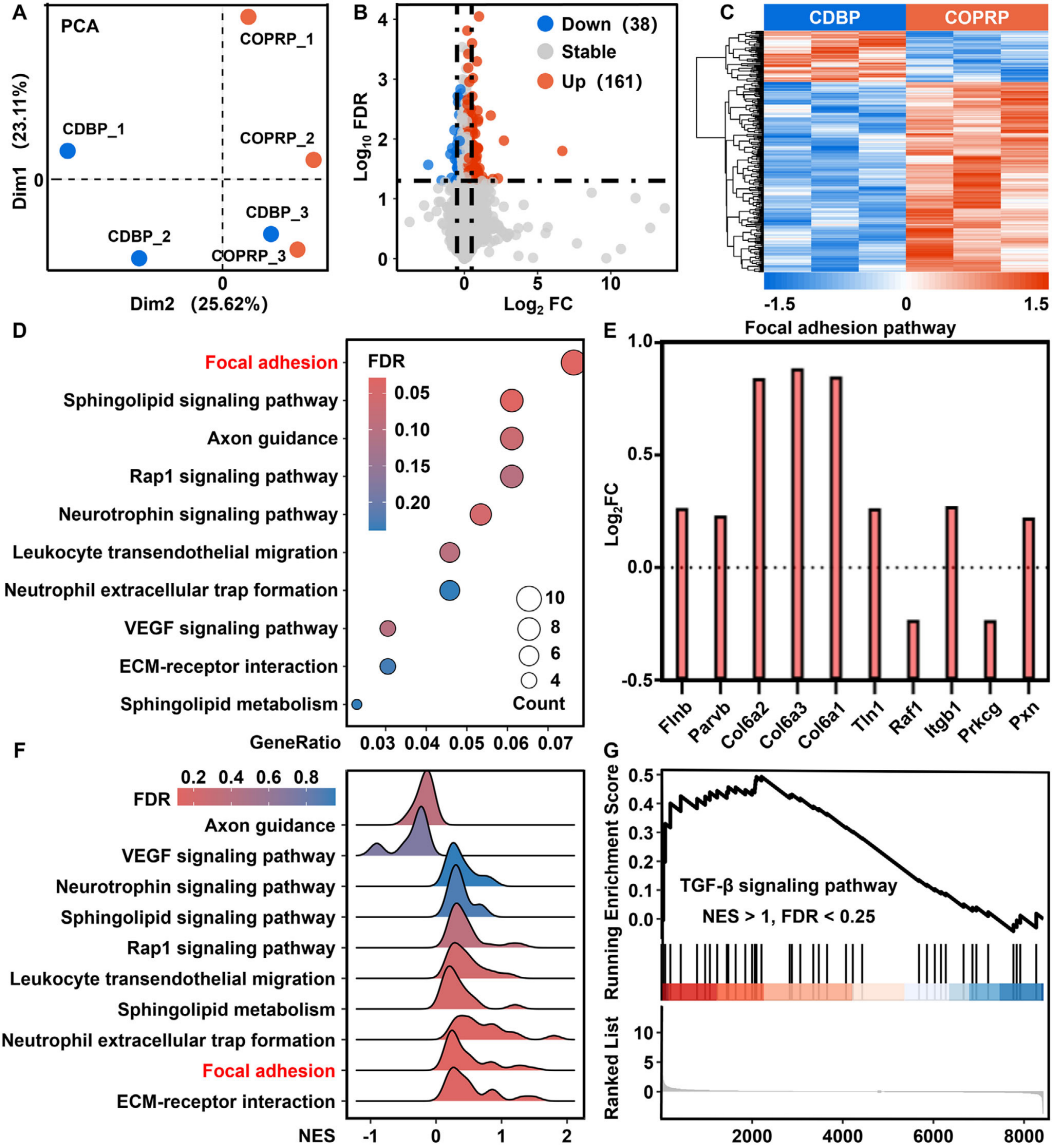
Proteomic analysis of brain tissue and validation of the TGF‐β/Smad2/3 signaling pathway. (A) Principal component analysis (PCA) of proteomic data from CDBP‐ and COPRP‐treated rat brain tissues (n = 3 per group), demonstrating clear inter‐group separation. (B) Volcano plot displaying differentially expressed proteins (DEPs) between the COPRP and CDBP groups; proteins with significant upregulation (161, orange) and downregulation (38, blue) are highlighted. (C) Heatmap of hierarchical clustering shows coordinated protein expression changes between the CDBP and COPRP groups. (D) Kyoto Encyclopedia of Genes and Genomes (KEGG) pathway enrichment bubble plot of DEPs; bubble size represents the number of enriched proteins, and color indicates the false discovery rate (FDR). (E) Bar chart of Log_2_ fold changes for representative DEPs enriched in the focal adhesion pathway. (F) Ridge plot illustrating the normalized enrichment score (NES) distribution across the top enriched KEGG pathways. (G) Gene set enrichment analysis (GSEA) plot confirming significant upregulation of the TGF‐β signaling pathway (NES > 1, FDR < 0.25) in COPRP‐treated brain tissue.

Furthermore, gene set enrichment analysis (GSEA) confirmed that all genes enriched within the TGF‐β signaling pathway exhibited significant upregulation (NES > 1, FDR < 0.25) (Figure 8F,G). The TGF‐β signaling pathway constitutes a highly conserved cellular communication system, extensively involved in regulating cell proliferation, differentiation, migration, apoptosis, and extracellular matrix synthesis, proving crucial in PD. It maintains a close bidirectional dialogue with focal adhesion, jointly coordinating cellular perception and response to the external microenvironment. In Figure S15, the expression levels of Smad2 and TGFβ2 proteins within the TGF‐β signaling pathway showed a marked increase, suggesting that COPRP function may regulate the transmission of the canonical Smad pathway.

### 3.8 Validation of COPRP Hydrogel Treatment for PD via TGF‐β/Smad Pathway

This study examined the potential mechanistic pathway identified by proteomics using WB analysis and pharmacological intervention. First, regarding correlation, the protein levels, i.e., WB, of relevant biomarkers were verified using brain tissue samples from PD rats in each group following conventional treatment, as shown in Figure 9A. The uncropped images for WB in Figure 9 are presented in Figures S16 and S17. WB analysis confirmed that COPRP treatment not only increased the protein abundance of total Smad2/3 but also markedly enhanced its phosphorylated form (p‐Smad2/3), signifying functional activation of this pathway (Figure 9B–E). Typically, within the pathological context of PD, elevated expression of the astrocyte marker GFAP directly indicates reactive proliferation, representing the brain's classic response to neuronal injury and inflammation. However, in this study, COPRP treatment unexpectedly and significantly reduced GFAP expression levels (Figure S18). This suggests COPRP intervention may fundamentally regulate the activation state of astrocytes. We hypothesize that active components in COPRP activate the TGF‐β/Smad pathway, thereby not merely suppressing astrocyte reactivity but guiding their transition from a chronic, potentially neurotoxic ‘hyperactivated’ phenotype toward a more protective and supportive ‘repair’ phenotype. A potential marker of this phenotypic shift is the reduction in GFAP expression, concurrent with enhanced capacity for synthesizing protective extracellular matrix components. Consequently, COPRP coordinates the functional output of astrocytes, proactively restructuring the pathological injury microenvironment into a protective microenvironment that supports neuronal survival and regeneration by activating TGF‐β signaling. Although the OPRP dosage administered in this study was notably low, existing research proposes that high fructose levels can promote TGF‐β activation in the treatment of inflammatory bowel disease [82], which echoes the findings of this study. Meanwhile, the OPRP prepared in this study contains a high proportion of fructans, especially inulin neoseries‐type. Existing research has demonstrated that these specific sugar units exert regulatory effects on the TGF‐β/Smad signaling pathway and apoptosis [83], although this has not been confirmed in neurological disorders.

**FIGURE 9:**
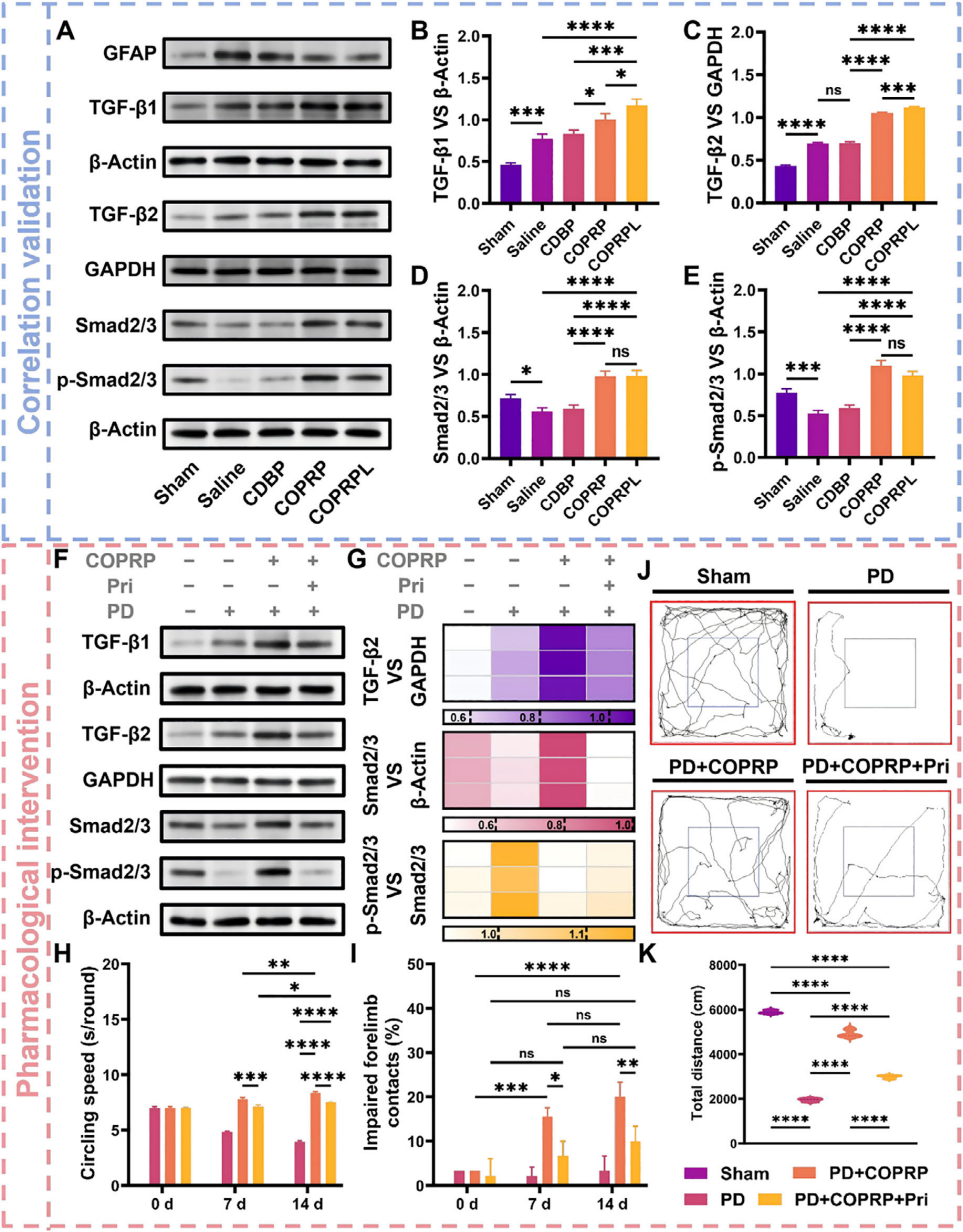
Validation of COPRP hydrogel treatment for PD via TGF‐β/Smad signaling pathway. Correlation validation: (A) Representative WB bands for GFAP, TGF‐β1, TGF‐β2, Smad2/3, p‐Smad2/3, GAPDH, and β‐Actin in rat brain tissue across all experimental groups (Sham, Saline, CDBP, COPRP, and COPRPL). (B) Quantification of TGF‐β1 protein expression normalized to β‐Actin. (C) Quantification of TGF‐β2 protein expression normalized to GAPDH. (D, E) Quantification of total Smad2/3 and phosphorylated p‐Smad2/3 protein expression normalized to β‐Actin. Pharmacological intervention: (F) Representative WB bands for TGF‐β1, TGF‐β2, Smad2/3, p‐Smad2/3, GAPDH, and β‐Actin in rat brain tissue from the pharmacological‐intervention groups (Sham, PD, PD+COPRP, and PD+COPRP+Pir). (G) Quantification of TGF‐β2 vs GAPDH, total Smad2/3 vs. β‐Actin, and p‐Smad2/3 vs. Smad2/3, reflecting pathway activation status across pharmacological‐intervention groups. Functional recovery evaluation in each pharmacological‐intervention group of PD rats was quantitatively assessed based on (H) the rotational asymmetry speeds and (I) the impaired forelimb contact proportion. As for the open field test, (J) total distance traveled and line crossing in the open field with the center zone were shown and (K) related quantification was counted through Smart 3.0 software. All WB data are presented as mean ± STD (n ≥ 3). ^*^p < 0.05, ^**^p < 0.01, ^***^p < 0.001, and ^****^p < 0.0001; ns, not significant.

Subsequently, an in vivo pharmacological intervention was conducted to further examine the TGF‐β‐related mechanism. Rats received intraperitoneal pretreatment with Pir (250 mg/kg), a pleiotropic antifibrotic agent reported to attenuate TGF‐β‐associated signaling [39], before 6‐OHDA lesioning and subsequent COPRP treatment. This design allowed us to ask whether the therapeutic benefits of COPRP are contingent on an intact TGF‐β/Smad axis, or whether they arise through parallel, TGF‐β‐independent mechanisms. However, several limitations of the Pir‐based pharmacological intervention should be acknowledged. Because Pir was administered systemically before 6‐OHDA lesioning, its effects were not restricted to striatal TGF‐β signaling, and potential influences on peripheral immune responses, systemic metabolism, hepatic and renal function, and PD model induction cannot be excluded. Therefore, our findings support this pathway as an important mediator of COPRP activity but do not establish pathway‐specific causality or exclude the involvement of alternative mechanisms. WB analysis of brain tissue from the pharmacological‐intervention groups, i.e., Sham, PD, PD+COPRP, and PD+COPRP+Pir, revealed a consistent and informative pattern (Figure 9F). Among the TGF‐β family members examined, TGF‐β2 exhibited a prominent response to COPRP treatment. TGF‐β2 is an important cytokine in the central nervous system and is expressed by multiple neural cell populations, including astrocytes and microglia. Unlike its well‐known role in peripheral fibrosis, accumulating evidence suggests that TGF‐β2 participates in maintaining neural homeostasis, regulating glial responses, controlling neuroinflammation, and promoting neuronal survival after injury through Smad‐dependent signaling. In the present study, COPRP treatment significantly increased TGF‐β2 expression and enhanced Smad2/3 phosphorylation compared with the PD group, consistent with the proteomic correlation analysis. Because TGF‐β1 is another major isoform of the TGF‐β family and shares highly conserved downstream TGFBR‐Smad2/3 signaling with TGF‐β2, we additionally examined TGF‐β1 expression to determine whether COPRP exerted broader regulation of the TGF‐β network. Interestingly, TGF‐β1 displayed a similar alteration trend to TGF‐β2 following COPRP treatment (Figure S19), suggesting coordinated activation of TGF‐β signaling rather than selective regulation of a single isoform. However, considering the prominent involvement of TGF‐β2 in CNS immune regulation and neuroprotective responses, TGF‐β2 was selected as the primary mechanistic focus of this study. Strikingly, co‐administration of Pir with COPRP substantially reversed these increases, suppressing both TGF‐β2 protein levels and pathway activation as reflected by the p‐Smad2/3/Smad2/3 ratio (Figure 9G). Similar conclusions were reached in the behavioral validation. Results from the rotational speed test and the cylinder test showed that the efficacy of the co‐administration of COPRP and Pir was significantly lower than that of COPRP treatment alone, but still far higher than that of the PD group, as demonstrated in Figure 9H. The open‐field test results presented in Figure 9J,K are also highly consistent with the previous pharmacological‐intervention results. Together, these findings indicate that COPRP‐induced activation of the TGF‐β2‐associated Smad signaling pathway contributes substantially to its neuroprotective effects. Pharmacological attenuation of TGF‐β signaling partially reversed the anti‐inflammatory and neuroprotective phenotype established by COPRP, supporting TGF‐β signaling as an important mediator in COPRP‐mediated protection against 6‐OHDA‐induced dopaminergic neuronal injury.

Mechanistically, these findings support a model in which bioactive components of OPRP, particularly its inulin neoseries‐type fructan components, promote activation of endogenous TGF‐β signaling within the injured SNc microenvironment. Enhanced TGF‐β2 signaling, together with coordinated regulation of TGF‐β1, activates the canonical Smad2/3 cascade and orchestrates downstream protective responses, including attenuation of excessive astrocyte activation, suppression of M1‐like microglial polarization and inflammatory cytokine production, and preservation of TH‐positive dopaminergic neurons. Inhibition of this signaling axis by Pir partially reversed these effects, further supporting the involvement of TGF‐β/Smad signaling in COPRP‐mediated neuroprotection. Collectively, these data support a mechanistic framework in which COPRP activates a neuroprotective TGF‐β2‐centered signaling network, highlighting the therapeutic potential of polysaccharide‐based biomaterials derived from traditional herbal medicine for PD treatment. In the future, we will introduce cell‐type‐specific TGF‐β/Smad signaling knockout models to precisely identify the cellular targets of COPRP‐mediated neuroprotection, thereby further strengthening the translational relevance of our findings.

Although the COPRP platform shows promise in preclinical treatment, this study was unable to establish clinical and pharmacoeconomic advantages over existing PD medications. Existing levodopa treatment regimens are clinically well‐established and easy to administer, whereas the current COPRP strategy requires stereotactic intracranial administration and specialized biomaterial preparation, which may increase surgical complexity and upfront costs. Any potential long‐term economic benefits will depend on whether the approach demonstrates sustained efficacy, reduced systemic drug exposure or dosing burden, acceptable manufacturing costs, and improved quality‐adjusted outcomes. These issues require direct comparison with optimized standard therapies, long‐term pharmacokinetic and safety studies, the development of less invasive delivery methods, and formal cost‐effectiveness analyses.

## 4 Conclusion

An injectable, self‐healing bioactive hydrogel (COPRP) was constructed by crosslinking the inulin neoseries‐type fructan OPRP with CMC via reversible Schiff base bonds. The optimized formulation exhibited a storage modulus of ∼230 Pa, uniform porosity with a mean pore diameter of ∼45.4 µm, injectability through a 30G needle, and sustained release of a hydrophilic model compound over 14 days. Departing from conventional hydrogel‐based drug delivery paradigms [84], this work establishes that the COPRP matrix itself, independent of any pharmacological payload, possesses intrinsic multi‐target biological activity rooted in the OPRP polysaccharide main chain. Drug‐free COPRP scavenged ROS, suppressed microglial M1 polarization by more than 50% across pro‐inflammatory markers while upregulating CD206 by over 3‐fold, restored mitochondrial membrane potential, attenuated apoptosis, and maintained TH expression in 6‐OHDA‐challenged dopaminergic neuron‐like cells. In the 6‐OHDA rat model, COPRP significantly ameliorated motor deficits, preserved TH‐positive dopaminergic neurons, attenuated glial activation, and elevated cerebral antioxidant capacity. The further loading of levodopa into COPRPL constitutes a deliberate dual‐mechanism therapeutic design: the intrinsic bioactivity of OPRP drives active remodeling of the pathological brain microenvironment, while levodopa loading provides complementary dopaminergic supplementation.

The potential involvement of TGF‐β/Smad signaling was examined using proteomic analysis, WB, and pharmacological intervention. Proteomic analysis of SNc tissue revealed broad upregulation of TGF‐β signaling pathway components, and subsequent WB quantification confirmed significant increases in TGF‐β1 and TGF‐β2 protein abundance, total Smad2/3, and p‐Smad2/3 in COPRP‐treated animals. In vivo pharmacological intervention with Pir, a pleiotropic antifibrotic agent reported to attenuate TGF‐β‐associated signaling, was performed prior to COPRP treatment. Pir co‐administration substantially reversed the neuroprotective, anti‐neuroinflammatory, and astrocyte‐modulatory phenotypes conferred by COPRP alone, and significantly attenuated behavioral recovery across rotational, cylinder, and open‐field tests. These findings support an important contribution of TGF‐β/Smad signaling to COPRP activity but do not establish pathway‐specific causality. Mechanistically, the inulin neoseries‐type fructan units within OPRP are proposed to enhance TGF‐β2‐associated signaling together with TGF‐β1 regulation in the lesioned substantia nigra microenvironment, activating canonical Smad2/3 phosphorylation and thereby coordinating downstream attenuation of reactive astrogliosis, suppression of M1‐biased microglial activation, and maintenance of dopaminergic neuron integrity. In contrast to recently reported PD hydrogel systems centered primarily on passive support or therapeutic‐cargo delivery, COPRP employs structurally defined OPRP as both a dynamic crosslinker and an intrinsically bioactive component. Drug‐free COPRP independently modulates oxidative stress, neuroinflammation, and dopaminergic neuronal injury, with TGF‐β/Smad2/3 signaling identified as an important mechanistic mediator. This structure–material–bioactivity–mechanism framework represents the principal advance of the present system, whereas levodopa loading provides complementary dopaminergic supplementation. Taken together, these findings position COPRP as a bioactive polysaccharide‐based therapeutic material rather than a passive drug depot. More broadly, this study provides a mechanistically grounded proof of concept that chemical functionalization of a structurally defined natural polysaccharide can couple injectable hydrogel formation with local regulation of oxidative stress, neuroinflammation, and dopaminergic neuronal injury. Further studies should evaluate long‐term biosafety, including hematological and serum biochemical indices, biodistribution, and the potential systemic effects of degradation products.

## Author Contributions

**Peng Dai**: investigation, writing – original draft, visualization, conceptualization. **Jikuang Zhao**: investigation, validation, methodology. **Chang Xue**: conceptualization, resources, investigation. **Kailei Xu**: methodology, validation. **Liang Yong**: methodology, validation. **Zhuangwei Zhang**: conceptualization, software. **Yi Huang**: conceptualization, formal analysis. **Jianwei Shuai**: writing – review and editing, funding acquisition, resources. **Xianzhen Chen**: resources, conceptualization, writing – review and editing. **Sheng Nie**: resources, methodology, writing – review and editing. **Junpeng Xu**: writing – review and editing, conceptualization, methodology, supervision, funding acquisition, project administration.

## Conflicts of Interest

The authors declare no conflicts of interest.

## Supporting information




**Supporting File**: advs77842‐sup‐0001‐SuppMat.docx.

## Data Availability

The data that support the findings of this study are available from the corresponding author upon reasonable request.
