# Supplementary material for: Modulation of the TGF‐β/Smad Signaling Pathway by a Bioactive Oxidized Polygonati Rhizoma Polysaccharide Crosslinked Chitosan Hydrogel as Therapeutic Carrier for Parkinson's Disease Treatment
Source: Adv Sci (Weinh). 2026 Sep 30:e77842. Online ahead of print. doi: 10.1002/advs.77842 (PMC13626671; doi:10.1002/advs.77842)
Supplement: Supplementary file 1 [file ADVS-9999-e77842-s001.docx]

**Supporting information**

**Modulation of the TGF-β/Smad signaling pathway by a bioactive oxidized *Polygonati rhizoma* polysaccharide crosslinked chitosan hydrogel as therapeutic carrier for Parkinson’s disease treatment**

Peng Dai ^1,2,3^, Jikuang Zhao ^4^, Chang Xue ^5^, Kailei Xu ^6^, Liang Yong ^7^, Zhuangwei Zhang ^1^, Yi Huang ^4^, Jianwei Shuai ^2,*^, Xianzhen Chen ^8,*^, Sheng Nie ^4,*^, Junpeng Xu ^1,4,9,*^

^1^ Central Laboratory of the Medical Research Center, the First Affiliated Hospital of Ningbo University, Ningbo, Zhejiang 315020, China

^2^ UCAS Wenzhou Institute, Wenzhou Medical University, Wenzhou, Zhejiang 325000, China

^3^ National Institute for Data Science in Health and Medicine, Xiamen University, Xiamen, Fujian 361102, China

^4^ Ningbo Key Laboratory of Nervous System and Brain Function, Neurosurgery Department, the First Affiliated Hospital of Ningbo University, Ningbo, Zhejiang 315000, China

^5^ State Key Laboratory of Macromolecular Drugs and Large-scale Preparation, School of Pharmaceutical Science, Wenzhou Medical University, Wenzhou, Zhejiang 325000, China

^6^ Center for Medical and Engineering Innovation, Central Laboratory, the First Affiliated Hospital of Ningbo University, Ningbo, Zhejiang 315010, China

^7^ Laboratory of Stem Cell, The First Affiliated Hospital of Ningbo University, Ningbo, Zhejiang 315010, China

^8^ Department of Neurosurgery, Shanghai Tenth People's Hospital of Tongji University, Shanghai 200072, China

^9^ Cixi People’s Hospital, Wenzhou Medical University, Ningbo, Zhejiang 315300, China

**Supporting Table: 4**

**Supporting Figures: 19**

***Corresponding authors:**

**Prof. Dr. Junpeng Xu:** Email: [fyxujunpeng@nbu.edu.cn](mailto:wangzhouguang@wmu.edu.cn)

**Prof. Dr. Sheng Nie:** Email: [fyyniesheng@nbu.edu.cn](mailto:wangzhouguang@wmu.edu.cn)

**Prof. Dr. Xianzhen Chen:** Email: chenxianzheny@126.com

**Prof. Dr. Jianwei Shuai:** Email: [shuaijw@wiucas.ac.cn](mailto:shuaijw@wiucas.ac.cn)

**Table S1.** Monosaccharide composition of OPRP.

| **Name** | **Glc** | **Fru** | **Ara** |
| --- | --- | --- | --- |
| Molar percentage of individual monosaccharides (%) | 5.85 | 93.88 | 0.28 |

**Table S2.** Methylation analysis results of OPRP.

| **Type of linkage** | **Methylated sugar** | **Retention time**  **(min)** | **Major mass fragments (m/z)** | **Mw** | **Relative molar ratio (%)** |
| --- | --- | --- | --- | --- | --- |
| t-Fru(f) | 2,5-di-O-acetyl-(2-deuterio)-1,3,4,6-  tetra-O-methyl hexitols (mannitol,glucitol) | 7.995 | 87,101,102,129,  161,162,205 | 323 | 26.08 |
| t-Glc(p) | 1,5-di-O-acetyl-2,3,4,6-tetra-  O-methyl glucitol | 9.521 | 87,102,118,129,  145,161,162,205 | 323 | 1.08 |
| 1-Fru(f) | 1,2,5-tri-O-acetyl-(2-deuterio)-3,4,6-  tri-O-methyl hexitols (mannitol,glucitol) | 13.080 | 87,101,129,145,  161,190 | 351 | 28.25 |
| 1-Fru(f) | 1,2,5-tri-O-acetyl-(2-deuterio)-3,4,6-  tri-O-methyl hexitols (mannitol,glucitol) | 13.203 | 87,101,129,145,  161,190 | 351 | 26.14 |
| 6-Glc(p) | 1,5,6-tri-O-acetyl-2,3,4-tri-  O-methyl glucitol | 14.455 | 87,99,102,118,  129,162,189,233 | 351 | 3.20 |
| 1,6-Fru(f) | 1,2,5,6-tetra-O-acetyl-(2-deuterio)-3,4-  di-O-methyl hexitols (mannitol, glucitol) | 18.999 | 87,99,100,129,  189,190 | 379 | 5.36 |
| 1,6-Fru(f) | 1,2,5,6-tetra-O-acetyl-(2-deuterio)-3,4-  di-O-methyl hexitols (mannitol, glucitol) | 19.190 | 87,99,100,129,  189,190 | 379 | 9.90 |

**Table S3.** Carbon-hydrogen signal assignment table of OPRP residues.

| **Residue Name** | **H1/C1** | **H2/C2** | **H3/C3** | **H4/C4** | **H5/C5** | **H6/C6** |
| --- | --- | --- | --- | --- | --- | --- |
| β-1,2-  Fru-(2→ | 3.65(3.94)/62.89 | -/103.28 | 4.20/76.76 | 4.06/74.46 | 3.88/81.63 | 3.65(3.88)/60.00 |
| β-t-  Fru-(2→ | 3.66(3.87)/60.02 | -/103.74 | 4.06/75.19 | 4.04/74.79 | 3.78/81.21 | 3.61(3.86)/60.08 |
| β-1,2,6-  Fru-(2→ | 3.66(3.89)/62.78 | -/104.78 | 4.05/74.96 | 4.03/76.75 | 3.77/81.16 | 3.66(3.89)/62.98 |
| α-t-  Glc-(1→ | 5.41/92.47 | 3.54/70.44 | 3.72/72.20 | 3.54/70.44 | 3.95/70.45 | 3.64(3.73)/60.34 |
| α-1,6-  Glc-(1→ | 4.92/88.40 | 3.78/72.19 | 3.69/72.35 | 3.55/70.98 | 3.92/71.41 | 3.73(3.99)/65.56 |

**Table S4.** Basic properties of the hydrogels with different formulas.

| **Sample** | **OPRP (wt%)** | **CMC (wt%)** | **Storage modulus (Pa)** | **Self-healing time (min)** | **Average pore diameter (μm)** |
| --- | --- | --- | --- | --- | --- |
| COPRP1 | 1.25 | 5 | ~59 | ~4 | 41.2 ± 15.9 |
| COPRP | 2.5 | 5 | ~230 | ~12 | 45.4 ± 4.9 |
| COPRP2 | 5 | 5 | ~834 | ~30 | 70.9 ± 21.9 |

**Fig S1.** The ^1^H-NMR spectra of PRP.

**Fig S2.** (A) SEM images of COPRP1 and COPRP2 hydrogels. (B) SEM pore size distribution statistics for the COPRP1/COPRP/COPRP2 hydrogel. All data are presented as mean ± STD (n ≥ 3). **p* < 0.05, ****p* < 0.001, and *****p* < 0.0001; ns, not significant.

**Fig S3.** Rheological data by amplitude sweep test of the COPRP hydrogel in the range of 1 to 560% dynamic strain amplitudes at 1 Hz frequency. Black arrows show the gel-to-sol points at ~370% strain.

**Fig S4.** Rheological data of COPRP1 and COPRP2 hydrogels. (A) Time-sweep experiment, (B) amplitude sweep test, (C) rheological self-healing test, and (D) steady shear rate test of the COPRP1 hydrogel. (E) Time-sweep experiment, (F) amplitude sweep test, (G) rheological self-healing test, and (H) steady shear rate test of the COPRP2 hydrogel.

**Fig S5.** Quantitative graph of cell viability in SH-SY5Y cells cultured with different concentrations of levodopa. All data are presented as mean ± STD (n ≥ 3). *****p* < 0.0001.

**Fig S6.** Quantification of ROS assay after LPS-induced BV2 microglia. All data are represented as mean ± STD (n ≥ 3). **p* < 0.05, ***p* < 0.01, and *****p* < 0.0001 between the indicated groups.

**Fig S7.** Photographs of the original staining bands and the composite whole-membrane images of CD206, IL-1β, and β-tubulin from the in vitro cellular anti-inflammatory WB experiment.

**Fig S8.** Photographs of the original staining bands and the composite whole-membrane images of iNOS, TNF-α, and β-tubulin from the in vitro cellular anti-inflammatory WB experiment.

**Fig S9.** Quantification of (A) IL-1β, (B) iNOS, (C) TNF-α, and (D) CD206 after LPS-induced BV2 microglia. All data are presented as mean ± STD (n ≥ 3). **p* < 0.05, ****p* < 0.001, and *****p* < 0.0001; ns, not significant.

**Fig S10.** Quantitative analysis of cell viability in 6-OHDA-induced SH-SY5Y cells across experimental groups. All data are presented as mean ± STD (n ≥ 3). **p* < 0.05, ***p* < 0.01, and *****p* < 0.0001.

**Fig S11.** Photographs of the original staining bands and the composite whole-membrane images of TH and GAPDH from the in vitro cellular PD models following each experimental group’s treatment.

**Fig S12.** The H&E staining images of SNc region for the COPRP-injected PD rat brain tissue-section after 7 or 14 days of treatment compared to the positive control Sham group and negative control Saline group after 14 days.

**Fig S13.** Representative H&E staining images of the heart, liver, spleen, lung, and kidney collected from rats in all experimental groups at the experimental endpoint. No apparent treatment-related histopathological abnormalities were observed.

**Fig S14.** Quantification of total superoxide dismutase (SOD) activity in SNc region of rat brain after treatment was investigated for in vivo antioxidative capability. All data are presented as mean ± STD (n ≥ 3). ****p* < 0.001 and *****p* < 0.0001.

**Fig S15.** Differentially expressed proteins in the TGF-β signaling pathway. Bar chart showing Log_2_ fold changes of all detected proteins enriched in the TGF-β signaling pathway in COPRP-treated versus CDBP-treated rat brain tissues. Red arrows highlight the markedly upregulated proteins Smad2 and TGF-β2. The dotted line indicates Log_2_ FC = 0.

**Fig S16.** The uncropped raw images of WB analysis including GFAP, TGF-β1, TGF-β2, Smad2/3, p-Smad2/3, GAPDH, and β-Actin for correlation validation of TGF-β signaling pathway from brain tissue.

**Fig S17.** The uncropped raw images of WB analysis including TGF-β1, TGF-β2, Smad2/3, p-Smad2/3, GAPDH, and β-Actin for casual validation of TGF-β signaling pathway from brain tissue.

.

**Fig S18.** Quantification of GFAP expression normalized to β-Actin. All data are represented as mean ± STD (n ≥ 3). *****p* < 0.0001 between the indicated groups.

**Fig S19.** Quantification of TGF-β1 expression normalized to β-Actin. All data are represented as mean ± STD (n ≥ 3).
